# Dissecting the Genetic Architecture of Phenology Affecting Adaptation of Spring Bread Wheat Genotypes to the Major Wheat-Producing Zones in India

**DOI:** 10.3389/fpls.2022.920682

**Published:** 2022-07-06

**Authors:** Pradeep Kumar Bhati, Philomin Juliana, Ravi Prakash Singh, Arun Kumar Joshi, Manish Kumar Vishwakarma, Jesse Poland, Velu Govindan, Sandesh Shrestha, Leonardo Crespo-Herrera, Suchismita Mondal, Julio Huerta-Espino, Uttam Kumar

**Affiliations:** ^1^International Maize and Wheat Improvement Center (CIMMYT), New Delhi, India; ^2^Borlaug Institute for South Asia (BISA), New Delhi, India; ^3^International Maize and Wheat Improvement Center (CIMMYT), Texcoco, Mexico; ^4^Department of Plant Pathology, Wheat Genetics Resource Center, Kansas State University, Manhattan, KS, United States; ^5^Campo Experimental Valle de México-INIFAP, Carretera los Reyes-Texcoco, Texcoco, Mexico

**Keywords:** heading, maturity, wheat, adaptation, GWAS, multi-environments

## Abstract

Spring bread wheat adaptation to diverse environments is supported by various traits such as phenology and plant architecture. A large-scale genome-wide association study (GWAS) was designed to investigate and dissect the genetic architecture of phenology affecting adaptation. It used 48 datasets from 4,680 spring wheat lines. For 8 years (2014–2021), these lines were evaluated for days to heading (DH) and maturity (DM) at three sites: Jabalpur, Ludhiana, and Samastipur (Pusa), which represent the three major Indian wheat-producing zones: the Central Zone (CZ), North-Western Plain Zone (NWPZ), and North-Eastern Plain Zone (NEPZ), respectively. Ludhiana had the highest mean DH of 103.8 days and DM of 148.6 days, whereas Jabalpur had the lowest mean DH of 77.7 days and DM of 121.6 days. We identified 119 markers significantly associated with DH and DM on chromosomes 5B (76), 2B (18), 7D (10), 4D (8), 5A (1), 6B (4), 7B (1), and 3D (1). Our results clearly indicated the importance of the photoperiod-associated gene (*Ppd*-B1) for adaptation to the NWPZ and the *Vrn*-B1 gene for adaptation to the NEPZ and CZ. A maximum variation of 21.1 and 14% was explained by markers 2B_56134146 and 5B_574145576 linked to the *Ppd*-B1 and *Vrn*-B1 genes, respectively, indicating their significant role in regulating DH and DM. The results provide important insights into the genomic regions associated with the two phenological traits that influence adaptation to the major wheat-producing zones in India.

## Introduction

Wheat (*Triticum aestivum* L.) is one of the most important food crops in the world, consumed by about 40% of the world’s population (Gupta et al., 2008). It provides about 20% of the daily protein and calories requirements for 4.5 billion people worldwide (Wright et al., 2021). Over 776.5 million tons of wheat was produced globally in 2021 (FAO, 2022). In India, wheat production touched a milestone output of 109.5 million tons in 2021 with a record nationwide average productivity of 3.42 t/ha.^1^ However, population growth, dietary changes, social and policy issues, the recent impact of COVID-19, and global unrest have increased the demand for staple foods such as wheat. Wheat yield is increasing by 0.9% per year, which is significantly lower than the required rate of ∼2.4% per year by 2050 (Ray et al., 2013). In addition, the present trends in wheat production are insufficient to feed the population of 9 billion people predicted for 2050 (Curtis and Halford, 2014; Aslam et al., 2022).

Without effective selection of adapted plants and genetic improvement, a global decrease of 6% in wheat production is estimated for 1°C increase in temperature (Zhao et al., 2017). The performance of a wheat variety is measured by its adaptability and yield potential under target environments, which is dependent on the genetic and environmental factors as well as the interaction between these factors. Wheat cultivation across the globe under different environmental and climatic conditions requires cultivar adaptability to a wide range of growing conditions. This adaptation is achieved by variations in phenology and related traits that affect plant architecture (Hyles et al., 2020). Wheat heading, flowering time, plant stature, and maturity time are important phenological and agronomic traits for adaptation, yield potential, and yield stability. The major genes affecting wheat adaptation include those associated with phenology and plant architecture, such as vernalization (*Vrn*), photoperiod (*Ppd*), earliness *per se* (*Eps*), and reduced height (*Rht*), in addition to other minor-effect loci.

Heading time is a vital component of wheat phenology under a complex genetic control. Timely heading is critical for the production to avoid late-season stresses, mainly terminal heat (Joshi et al., 2007). However, this stress can manifest itself at an early stage (Kumar et al., 2021). Three gene groups, including *Vrn*, *Ppd*, and *Eps*, have a major influence on heading and flowering time of wheat (Snape et al., 2001; Distelfeld et al., 2009; Kumar et al., 2012; Khumalo et al., 2017). Early studies reported that the switch from vegetative to reproductive development is promoted by the prolonged cold temperatures of winter (vernalization) (Chouard, 1960). Unlike winter types, spring wheats require little-to-no environmental inducement for flowering. Vernalization requirement is typically combined with day-length-responsive flowering, such that plants that have vernalized over winter will flower rapidly as days subsequently lengthen in spring (Chouard, 1960). Vernalization occurs most rapidly at 4.9°C and requires temperatures between −1.3 and 15.7°C (Porter and Gawith, 1999).

The vernalization requirement of wheat is controlled by the *Vrn*1 genes located on the long arm of chromosomes 5A, 5B, and 5D (Snape et al., 2001). The *Vrn*1 gene is the wheat ortholog of the meristem identity gene *APETALA1* from *Arabidopsis thaliana* and plays an important role in floral development by triggering early flowering when expressed at high levels (Yan et al., 2003). It is expressed in both leaves and shoots, and the accumulation of *Vrn*1 transcripts in the shoot apex is associated with the switch to reproductive development. The vernalization-insensitive alleles of *Vrn*1 (*Vrn*-A1, *Vrn*-B1, and *Vrn*-D1) shorten both the vegetative and the reproductive/maturity stages (Snape et al., 2001). The *Vrn*2 gene is a repressor of flowering that plays a key role in blocking the long-day flowering response before winter (Yan et al., 2004). The third gene (*Vrn*3), which can reduce the vernalization requirement of wheat, was identified to be completely linked to a gene similar to the *Arabidopsis FLOWERING LOCUS T* (Yan et al., 2006). The *Vrn*4 found in ancient wheat subspecies accessions from South Asia is located on chromosome 5DS and plays a role in local adaptation (Kippes et al., 2015).

Spring wheat can also have varying levels of sensitivity to day length. Day-length-insensitive spring cultivars can progress to the terminal spikelet stage and flower rapidly, even on short days. Photoperiod sensitivity to day length is largely determined by alleles of the photoperiod-1 (*Ppd*-1) genes located on chromosomes 2A, 2B, and 2D (Keim et al., 1973). Plants having an increased copy number of *Ppd*-B1 and *Vrn*-A1 alleles (*Vrn*-A1, *Vrn*-B1, and *Vrn*-D1) flowered early and showed increased vernalization requirement, respectively, suggesting that copy number variation is important for the adaptation of wheat (Díaz et al., 2012). Alleles of *Ppd*-D1 (PPD-D1a, PPD-B1a, and PPD-A1a) that confer a strong insensitivity to day length are associated with rapid flowering under all day-length conditions (Wilhelm et al., 2009; Díaz et al., 2012; Bentley et al., 2013). Alleles of *Ppd*-1 that are associated with reduced day-length sensitivity are also associated with an increased rate of spikelet development and decreased spike fertility (Prieto et al., 2018). A recent study attributed a shorter duration of pre-anthesis stem elongation and a decreased number of fertile florets to *Ppd*-D1a, highlighting the scope for increased yield potential by selection for photoperiod-sensitive alleles (Pérez-Gianmarco et al., 2019). Genes that influence the duration of the wheat life cycle under conditions where vernalization and photoperiod requirements have been met are described as “Earliness *per se*” (*Eps*) genes (Snape et al., 2001). Ochagavía et al. (2019) reported that allelic differences at the *Eps-D1* gene on chromosome 1D conferred differing levels of sensitivity to temperature; earliness was associated with an increased sensitivity to temperature during the late reproductive phase of development in hexaploid wheat.

Understanding the genetic basis of phenology and other adaptive traits in spring bread wheat is important for developing varieties adapted to various environmental conditions and stresses. In this regard, a genome-wide association study (GWAS) is a high resolution and an approach to dissect the genetic basis of complex traits. It is preferred over linkage mapping because it accounts for greater allelic diversity at a given locus and exploits the ancestral recombination events that have occurred in an existing diversity panel (landraces, elite cultivars, and advanced breeding lines) at the population level (Yu and Buckler, 2006; Zhu et al., 2008; Ingvarsson and Street, 2011; Waugh et al., 2014; Scherer and Christensen, 2016). Another advantage of GWAS is that they use available germplasm and bypass the time-consuming process of developing segregating populations. Moreover, QTL mapping by bi-parental populations focuses on specific traits, whereas a wider range of germplasm can be used in GWAS to phenotype many traits with one cycle of genotyping (Li et al., 2019). GWASs are more efficient and require less effort in analyzing complex traits under various environmental conditions (George and Cavanagh, 2015). They have been used successfully to dissect several complex traits in wheat (Breseghello and Sorrells, 2006; Crossa et al., 2007; Yu et al., 2011; Maccaferri et al., 2015; Sukumaran et al., 2015; Juliana et al., 2019, 2021). While few GWASs in wheat have identified genomic regions associated with phenological traits including heading and maturity (Zhang et al., 2018; Gahlaut et al., 2021; Muhammad et al., 2021), a comprehensive study to dissect the genetic architecture of these traits in multiple wheat production zones has not been reported. A total of 43 SNPs (single-nucleotide polymorphisms) were consistently detected, including seven across multiple environments by ML-GWAS (Muhammad et al., 2021). Nine significant marker–trait associates were identified for days to anthesis under drought stress by Gahlaut et al. (2021). Hence, the main objective of this study was to use GWAS to identify consistently significant marker–trait associations for heading and maturity, affecting the adaptation of spring bread wheat to three major zones of India where wheat is cultivated in about 25 mha under diverse environmental and management conditions.

## Materials and Methods

### Populations and Field Experimental Design

A total of 4,680 advanced breeding lines from eight South Asia Bread Wheat Genomic Prediction Yield Trial (SABWGPYT) panels were evaluated in eight consecutive crop cycles between 2014 and 2021. In each crop cycle, about 540–600 different genotypes including six high-yielding checks were planted in two replications at the research stations of the Borlaug Institute for South Asia (BISA) in the three main wheat-growing regions of India: (1) Ludhiana, Punjab [30° 59′ N, 75° 44′ E, representing the North-Western Plain Zone (NWPZ)], (2) Jabalpur, Madhya Pradesh [23° 22′ N, 80° 07′ E, representing the Central Zone (CZ)], and (3) Pusa, Samastipur, Bihar [25° 95′ N, 85° 66′ E, representing the North-Eastern Plain Zone (NEPZ)]. The panels were named by the site where they were evaluated followed by the harvest year (e.g., Jabalpur panel, 2014; Ludhiana panel, 2014; Pusa panel, 2014).

The lines were planted in an alpha lattice design (Patterson et al., 1978) during the optimum planting time (the second to third week of November at each location), and they received an optimum irrigation of about 500 mm of water in total from five or more irrigations. The whole experiment was divided into 10 trials with 60 entries per trial, except panel 2014 and panel 2021 (nine trials with 60 entries each). Each replicate was divided into six sub-blocks of 10 plots. The plot size was 5.016 m^2^, and the lines were sown in six rows, 22 cm apart and 3.8 m in length. Field trials were managed by standard agronomic practices recommended for the location.^2^ Fertilizer was applied as 150 N/60 P/40 K kg ha^–1^ at Ludhiana and 120 N/60 P/40 K kg ha^–1^ at Jabalpur and Pusa as per wheat-growing zone recommendation.

### Phenotyping and Statistical Analysis of the Phenotyping Data

The lines at all the sites were evaluated for Days to heading (DH) and days to maturity (DM) using alpha lattice design of field experiment. DH are counted visually as the number of days from planting until 50% of the spikes are visible through the flag leaf sheath. Similarly, DM was recorded as the number of days from planting to the day when 50% of the main tiller peduncles became entirely visible. In each of the 24 environments (three sites and eight panels), the best linear unbiased estimates (BLUEs) for DH and DM were obtained across replications using the META-R v6.03 software (Alvarado et al., 2020). The DH and DM were used as random effects while performing the analysis. Visualization of the trait distributions was performed using the “R” package “ggplot2” (Wickham, 2009).

For the two traits, we also obtained the following statistical measures using the META-R v6.03 software: (i) broad-sense heritabilities across replications to understand the selection response, (ii) coefficients of variation for each dataset to understand whether the field trial was well conducted, (iii) the least significant differences between genotypes for comparison and selection of the best genotypes, (iv) variance components including the genotypic variance and residual variance within each dataset to understand what proportion of the variance can be attributed to the genotypes, (v) genotypic and phenotypic correlations across sites, (vi) biplot based on the genetic correlations showing the relationship between the sites, and (vii) the variance components to understand whether there was a significant difference between the genotypes and sites. Heritability (h2) was estimated following Nyquist (1991) as h2 = 1 − [MS (Genotype × Year)]/MS (Genotype).

### Genotyping

Genotyping data for all the eight SABWGPYT panels used in this study were obtained using the genotyping-by-sequencing (GBS) method (Poland et al., 2012). We estimated the SNPs using the Trait Analysis by Association Evolution and Linkage (TASSEL) version 5 GBS pipeline (Glaubitz et al., 2014). This was followed by SNP discovery at a minor allele frequency of 0.01, and the resulting GBS tags were aligned to the reference sequence of bread wheat version 1.0 (RefSeq v1.0) using Bowtie (Langmead and Salzberg, 2012). The tags were then filtered using *p*-values from Fisher’s exact test, inbred coefficient, and chi-square as described in Juliana et al. (2021), and over 80,000 SNPs that passed at least one of these filters were obtained. We removed the markers that had greater than 50% missing data, less than 5% minor allele frequency, and greater than 10% heterozygosity. In addition, we also removed the lines with greater than 90% missing data in each panel. We obtained the following number of filtered markers and lines for GWAS: (i) panel 2014: 481 lines and 18,351 markers, (ii) panel 2015: 582 lines and 17,764 markers, (iii) panel 2016: 583 lines and 17,094 markers, (iv) panel 2017: 529 lines and 17,620 markers, (v) panel 2018: 540 lines and 16,152 markers, (vi) panel 2019: 528 lines and 16,771 markers, (vii) panel 2020: 540 lines and 16,709 markers, and (viii) panel 2021: 491 lines and 17,851 markers. Marker imputation was performed using Beagle version 4.1 (Browning and Browning, 2016).

### Genome Wide Association Mapping

We performed GWAS for DH and DM in all the 24 datasets for each trait using the mixed linear model (Yu et al., 2006) in TASSEL version 5 (Bradbury et al., 2007). The model was fitted using population structure as a fixed effect and kinship as a random effect, which were accounted for using the first two principal components (Patterson et al., 2006; Price et al., 2006) and the genomic relationship matrix obtained using the centered identity-by-state method (Endelman and Jannink, 2012), respectively. In addition, we used the optimum level of compression and the “population parameters previously determined” (Zhang et al., 2010) options for fitting the mixed linear model. We obtained the *p*-values for the tests of significance of the marker and trait associations, the additive effects of the markers, and the percentage of variation explained by them. The GWAS results were visualized using Manhattan plots using the “R” package “CMplot” (Lilin, 2018). Correction for multiple testing was performed using the Bonferroni correction at an α level of 0.20, and the markers which had *p*-values lower than the cut-off level (ranging between 1.09E-05 and 1.24E-05 in the different panels) were considered to be significant. In addition, we also obtained markers that were (i) consistently significant in more than one site that could indicate the markers associated with broad adaptation to different sites and (ii) associated with DH and maturity, which could provide insights into the genomic regions that were associated with both these phenological traits. The markers that were significant in more than one dataset were then plotted on a reference map using ‘‘Phenogram.’’^3^

## Results

### Phenotyping Data

**FIGURE 1:**
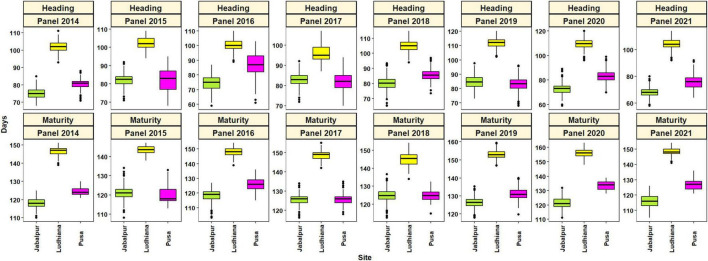
Boxplots showing the distributions of heading and maturity in Jabalpur, Ludhiana, and Pusa in panels 2014–2021.

Large and continuous variation was observed for the phenological traits in all three environments from 2014 to 2021 for the 4,680 advanced breeding lines (Table 1 and Figure 1). The DH (58–98 days) and DM (103–137 days) were shortest at Jabalpur, but longest (87–120 for DH and 138–163 for DM) at Ludhiana. The mean DH ranged from 96 (panel 2017) to 112 (panel 2018) days at Ludhiana, 68 (panel 2021) to 84 (panel 2019) days at Jabalpur, and 76 (2021) to 87 (2016) days at Pusa. For DM, the rage was 143 (panel 2015) to 156 (panel 2020) days at Ludhiana, 116 (2021) to 126 (2020) days at Jabalpur, and 120 (2015) to 134 (2020) days at Pusa.

**TABLE 1:** Descriptive statistics and variance parameters estimated for days to heading (DH) and maturity (DM) agronomic traits based on best linear unbiased estimate (BLUE) values in association panel grown at three different environments in India from 2014 to 2021.

Stat.	2013–2014						2014–2015					
		
	DTHD			DAYSMT			DTHD			DAYSMT		
				
	LDH	JBL	PUS	LDH	JBL	PUS	LDH	JBL	PUS	LDH	JBL	PUS
Range	90–111	67–85	65–88	139–151	110–125	121–130	94–109	71–98	68–95	138–147	108–134	113–133
Mean	102	75	81	146	118	124	102	82	82	143	121	120
H^2^ (%)	0.87	0.91	0.72	0.56	0.76	0.44	0.92	0.96	0.84	0.83	0.96	0.82
CV%	1.33	1.63	2.25	1.19	1.32	0.98	1.05	1.14	4.13	0.61	0.66	1.94
LSD%	2.65	2.52	3.06	2.69	2.92	2.62	2.15	1.89	6.51	1.68	1.66	4.48
σ2G	7.74	8.23	5.13	2.2	4.7	1.05	7.67	11.91	35.35	2.06	11.45	13.32
σ2E	1.98	1.61	3.78	3.16	2.49	1.63	1.17	0.92	12.24	0.84	0.78	5.52
	**2015–2016**						**2016–2017**					
Range	89–110	59–87	61–103	139–154	103–127	115–136	87–107	66–92	70–94	142–155	116–134	118–135
Mean	101	75	87	148	119	126	96	83	82	149	126	126
H^2^ (%)	0.95	0.98	0.89	0.83	0.88	0.81	0.95	0.9	0.79	0.81	0.86	0.78
CV%	1	1.1	3.99	0.8	1.45	2.01	1.04	1.51	2.77	0.81	0.94	1.32
LSD%	2.09	1.73	6.87	2.33	3.42	4.9	2.08	2.36	3.89	2.36	2.4	3.03
σ2G	12.42	24.52	55.33	3.91	12.33	15.42	11.97	8.66	14.57	3.54	5.11	5.64
σ2E	1.06	0.72	12.55	1.43	3.03	6.67	1.02	2.58	6.02	1.5	1.43	2.83
	**2017–2018**						**2018–2019**					
Range	94–115	65–93	74–97	134–154	112–137	110–132	102–120	73–98	68–96	147–159	118–135	120–140
Mean	105	80	86	145	125	125	112	84	83	153	126	131
H^2^ (%)	0.96	0.76	0.87	0.87	0.78	0.68	0.95	0.85	0.88	0.89	0.68	0.57
CV%	0.91	2.75	1.83	1.02	1.54	1.73	0.79	2.35	2.4	0.63	1.44	1.78
LSD%	2.03	3.78	3.05	2.91	3.51	3.62	1.9	3.93	3.87	1.94	3.17	3.46
σ2G	11.97	12.32	9.37	8.62	7.94	5.59	9.1	13.23	17.08	4.17	4.04	4.84
σ2E	0.94	6.22	2.85	2.29	3.87	4.99	0.8	4.07	4.42	0.95	3.42	5.74
	**2019–2020**						**2020–2021**					
Range	96–120	59–89	70–99	148–163	111–132	128–139	92–114	58–80	64–92	141–154	105–126	121–134
Mean	109	73	83	156	121	134	104	68	76	148	116	127
H^2^ (%)	0.94	0.95	0.85	0.87	0.87	0.76	0.92	0.94	0.95	0.77	0.9	0.82
CV%	1.01	1.58	2.71	0.66	1.36	1.21	1.06	1.58	2.06	0.76	1.46	1.35
LSD%	2.33	2.44	4.17	2.11	3.32	3.38	2.3	2.28	3.23	2.16	3.47	3.23
σ2G	11.07	15.24	18.56	4.07	11.85	4.96	10.34	10.41	28.09	2.28	14.5	7.96
σ2E	1.24	1.36	5.71	1.1	2.84	2.78	1.44	1.16	2.43	1.29	2.89	3.03

*DTHD, days to heading; DAYSMT, days to maturity; H^2^, heritability; CV, coefficient of variation; LSD, least significant difference; σ2G, genotypic variance; σ2E, residual variance; BLUE, best linear unbiased estimates; LDH, Ludhiana; JBL, Jabalpur; PUS, Pusa.*

We observed high heritability for DH over years, ranging from 0.87 to 0.98 at Ludhiana, 0.76 to 0.98 at Jabalpur, and 0.72 to 0.95 at Pusa (Table 1). Heritability for DM was moderate to high, ranging from 0.56 to 0.89 at Ludhiana, 0.68 to 0.96 at Jabalpur, and 0.44 to 0.82 at Pusa. The coefficient of variation ranged from 0.79 (Ludhiana panel 2019) to 4.13 (Pusa panel 2015) for DH and 0.61 (Ludhiana panel 2015) to 2.01 (Pusa panel 2016) for DM (Table 1).

### Phenotypic and Genetic Correlations for Days to Heading and Maturity Across Locations

For DH, the phenotypic Pearson correlations across locations ranged from 0.46 (panel 2017 between Pusa and Ludhiana) to 0.84 (panel 2021 between Pusa and Jabalpur) (Table 2). Similarly, for DM, it ranged from 0.31 (panel 2014 between Pusa and Ludhiana) to 0.72 (panel 2019 between Pusa and Jabalpur). Positive phenotypic correlations ranged from 0.32 to 0.85 between DH and DM between locations. We also observed moderate-to-strong genetic correlations ranging from 0.56 (panel 2017 between Pusa and Jabalpur) to 0.92 (panel 2020 between Pusa and Jabalpur) for DH and from 0.45 (panel 2021 between Pusa and Ludhiana) to 0.83 (panel 2020 between Pusa and Jabalpur) for DM (Table 3). Dendrograms and biplots showing the genetic correlations between different traits in different locations and years were obtained (Supplementary File 1).

**TABLE 2:** Phenotypic correlations between locations for days to heading (DH) and days to maturity (DM) from 2014 to 2021.

Years	Traits	LDH–JBL	LDH–PUS	JBL–PUS
2013–2014	DTHD	0.68	0.65	0.73
	DAYSMT	0.41	0.32	0.44
2014–2015	DTHD	0.74	0.62	0.68
	DAYSMT	0.54	0.53	0.61
2015–2016	DTHD	0.75	0.66	0.80
	DAYSMT	0.65	0.59	0.66
2016–2017	DTHD	0.49	0.46	0.46
	DAYSMT	0.65	0.67	0.59
2017–2018	DTHD	0.68	0.56	0.69
	DAYSMT	0.62	0.50	0.56
2018–2019	DTHD	0.55	0.47	0.50
	DAYSMT	0.69	0.68	0.72
2019–2020	DTHD	0.64	0.61	0.84
	DAYSMT	0.46	0.46	0.64
2020–2021	DTHD	0.62	0.69	0.85
	DAYSMT	0.40	0.37	0.65

*Significance at 0.01 probability level.*

*DTHD, days to heading; DAYSMT, days to maturity; LDH, Ludhiana; JBL, Jabalpur; PUS, Pusa.*

**TABLE 3:** Genetic correlations between locations for days to heading (DH) and days to maturity (DM) from 2014 to 2021.

Years	Traits	LDH–JBL	LDH–PUS	JBL–PUS
2013–2014	DTHD	0.76	0.81	0.91
	DAYSMT	0.73	0.62	0.68
2014–2015	DTHD	0.79	0.71	0.75
	DAYSMT	0.61	0.66	0.70
2015–2016	DTHD	0.78	0.71	0.85
	DAYSMT	0.75	0.72	0.79
2016–2017	DTHD	0.62	0.62	0.57
	DAYSMT	0.71	0.76	0.69
2017–2018	DTHD	0.78	0.62	0.84
	DAYSMT	0.73	0.66	0.77
2018–2019	DTHD	0.73	0.60	0.72
	DAYSMT	0.78	0.75	0.84
2019–2020	DTHD	0.69	0.69	0.93
	DAYSMT	0.53	0.56	0.78
2020–2021	DTHD	0.68	0.73	0.90
	DAYSMT	0.48	0.45	0.75

*Significance at 0.01 probability level.*

*DTHD, days to heading; DAYSMT, days to maturity; LDH, Ludhiana; JBL, Jabalpur; PUS, Pusa.*

The variance components of the traits showed that for both DH and DM, the genotypes and sites were significantly different in all the panels (Table 1).

### Genotyping Data and Population Structure Analysis

**FIGURE 2:**
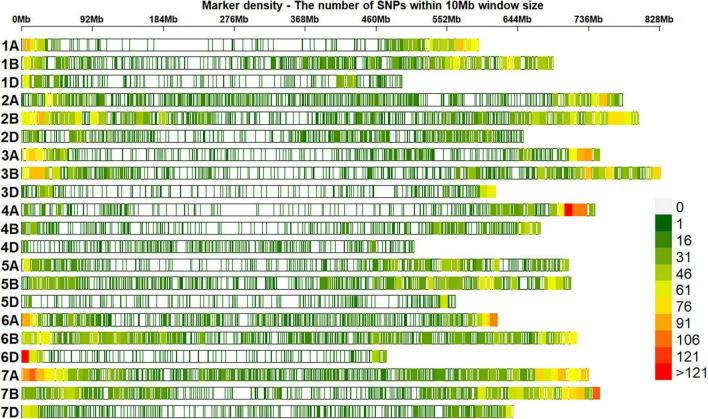
Densities of 23,581 genotyping-by-sequencing (GBS) single-nucleotide polymorphisms (SNPs) in the reference bread wheat genome (RefSeq v1.0). The color key with marker densities indicates the number of markers within a window size of 10 Mb.

**FIGURE 3:**
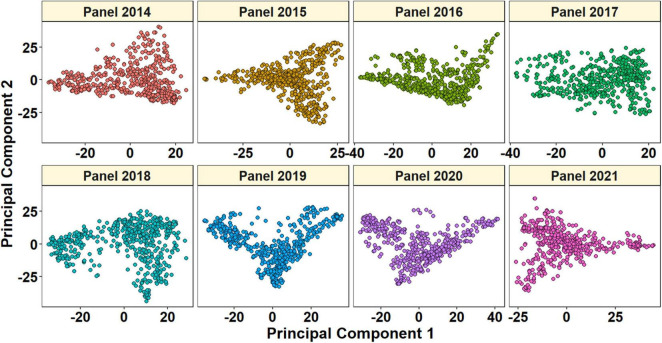
Plot of principal components 1 and 2 indicating the population structure in panels 2014–2021.

Of 23,979 unique GBS markers used in all the panels for GWAS, 23,581 were mapped to the RefSeq v1.0 and their densities within a window size of 10 Mb were obtained (Figure 2). We observed a good coverage of markers on all chromosomes with a high density on the telomeric end of chromosomes such as 4AL, 6A, 6DS, 7AS, and 7BS. The highest number of markers was on chromosomes 7A (2,227 markers) and 2B (2,148 markers), but the lowest on chromosomes 5D (296 markers), 4D (401 markers), 6D (445 markers), 1D (453 markers), and 3D (454 markers). A plot of the first two principal components in the eight panels indicated a weak-to-moderate population structure (Figure 3). The% variation explained by principal component analysis in each panel from 2014 to 2021 is given in Table 4.

**TABLE 4:** Principal component analysis (PCA) of the SABWGPTY panels obtained based on single-nucleotide polymorphism (SNP) genotyping.

	% Variation explained by PC1	% Variation explained by PC2
Panel 2014	8.81	5.68
Panel 2015	8.01	6.3
Panel 2016	11.2	4.6
Panel 2017	7.46	4.47
Panel 2018	11.77	8.59
Panel 2019	12.09	8.56
Panel 2020	12.77	7.65
Panel 2021	11.44	5.58

### Genome Wide Association Mapping for Days to Heading and Days to Maturity

We performed GWAS for DH and DM using 48 datasets and obtained the *p*-values, additive effects, and the percentage variation explained by each of the markers in the different panels (Supplementary File 2). After Bonferroni’s correction for multiple testing, we obtained 222 markers that were associated with the traits in the different datasets (Supplementary File 3). Among them, 119 markers were significant in at least two datasets and were considered as consistent significant markers (Supplementary File 4). The highest numbers of these markers were on chromosome 5B (76). The markers significantly associated with DH and DM in the different sites and panels are described in the following.

### Markers Significantly Associated With Days to Heading

In Jabalpur, among the 101 markers significantly associated with DH, 43 markers (43%) were consistently significant in more than one panel, and they were all on chromosome 5B, between 556185926 and 596948341 bps. The consistent markers had additive effects ranging between 1 and 1.95 days and explained 4.3–12.3% of the variation. Among them, marker 5B_574145576 was significant in the highest number of panels (panels 2016, 2018, 2019, 2020, and 2021), with additive effects ranging between 1.45 and 1.95 days and explained 7–12.3% of the phenotypic variation. In addition, 58 markers were significantly associated in only one panel in Jabalpur, including 38 markers on chromosome 5B between 447567055 and 689895400 bps, eight markers on chromosome 6B between 185171994 and 229054418 bps, five markers on chromosome 7D between 55486522 and 58633321 bps, and markers 2D_82143828, 3B_777688779, 3D_562906853, 5A_570528289, 5A_580935013, 7A_266640411, and 7B_669973991.

In Ludhiana, among the 77 markers significantly associated with DH, only seven markers (9.1%) were consistently associated in more than one panel which were all on chromosome 2B, between 53667024 and 59584538 bps. These consistent markers had additive effects ranging between 0.7 and 2.1 days and explained 4.6–21.1% of the phenotypic variation. Among them, marker 2B_56134146 was significant in the highest number of panels (panels 2015, 2017, 2018, 2019, and 2021), with additive effects ranging between 0.95 and 2.1 days and explained 4.9–21.1% of the phenotypic variation. We also observed that 70 markers were significantly associated with DH in Ludhiana in one dataset only, which included 27 markers on chromosome 2B, 14 markers on chromosome 7B, 11 markers on chromosome 4D, 10 markers on chromosome 7D, four markers on chromosome 5B, and markers 3A_21112167, 3D_56844252, 4A_684814200, and 7A_174693955.

In Pusa, among the 88 markers significantly associated with DH, 68 markers (77.3%) were consistently associated in more than one panel which were all on chromosome 5B between 549777627 and 596948341 bps. These consistent markers had additive effects ranging between 1 and 2.8 days and explained 4.3–14.1% of the phenotypic variation. We also observed that six markers (5B_574145576, 5B_575270556, 5B_576348143, 5B_586610468, 5B_586805570, and 5B_586862827) were significant in five panels and they had additive effects ranging between 1.2 and 2.7 days and explained 4.5–14.1% of the variation. In addition, 20 markers were significantly associated with DH in one panel only in Pusa, including 16 markers on chromosome 5B between 549730988 and 610372912 bps and markers 5A_481889948, 5A_580935013, 6A_559740187, and 7B_653976952.

### Markers Significantly Associated With Days to Maturity

In Jabalpur, among the 67 markers significantly associated with DM, only five markers (7.5%) were consistently significant in more than one panel. This included markers 5B_574145576, 5B_575270556, 5B_592792409, 5B_594614262, and 5B_594913947 that had additive effects ranging between 1 and 1.8 days and explained 4.5–9.5% of the phenotypic variation. Marker 5B_574145576 was significant in the highest number of panels (panels 2018, 2020, and 2021) and had additive effects ranging between 1.4 and 1.75 days and explained 4.6–9.5% of the phenotypic variation. Furthermore, the 62 markers were associated with DM in Jabalpur in one panel only, which included 36 markers on chromosome 5B between 550910513 and 595649946 bps, nine markers on chromosome 7D, eight markers on chromosome 6B, two markers on chromosomes 7A and 7B each, and five markers on other chromosomes.

In Ludhiana, among the 49 markers significantly associated with DM in the different panels after correction for multiple testing, only one marker (2%) was consistently significant in more than one panel. This marker (2B_56134146) had additive effects ranging between 0.6 and 1.4 days and explained 4.4–11.4% of the phenotypic variation. The 48 markers were significantly associated with DM in one panel only in Ludhiana, which included 18 markers on chromosome 2B, 11 markers on chromosome 7A, eight markers on chromosome 4D, five markers on chromosome 7D, two markers each on chromosomes 3A and 3D, and markers 1D_189345725 and 5B_601867021. In Pusa, among the 11 markers significantly associated with DM in the different panels after correction for multiple testing, only one marker (9%) was consistently significant in more than one panel. This marker (5B_574145576) had additive effects ranging between 0.95 and 1.1 days and explained 4.7–6.4% of the phenotypic variation. In addition, we also observed that 10 markers were significantly associated with DM in Pusa in one panel only.

### Markers Significantly Associated With Days to Heading in More Than One Site

**FIGURE 4:**
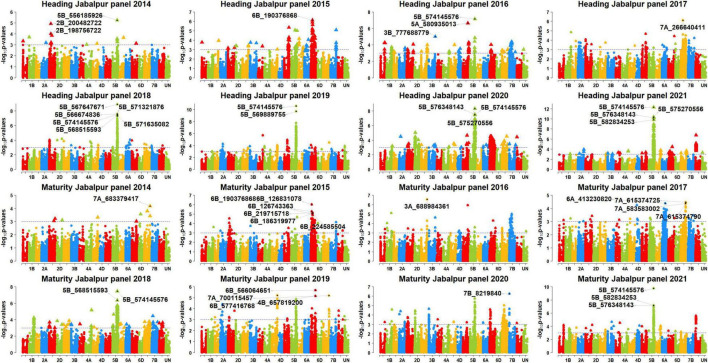
Manhattan plots showing the genomic regions significantly associated with days to heading (DH) and maturity (DH) in Jabalpur in panels 2014–2021.

**FIGURE 5:**
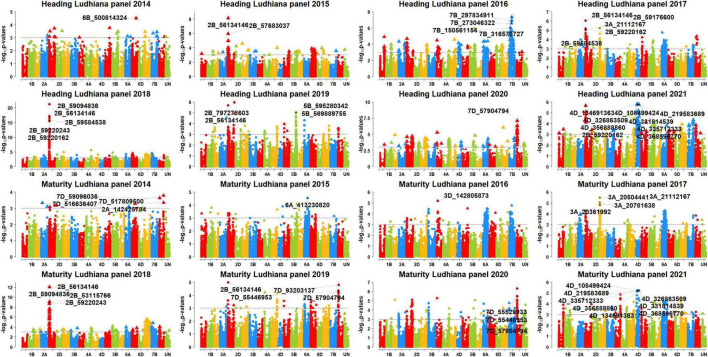
Manhattan plots showing the genomic regions significantly associated with days to heading (DH) and maturity (DM) in Ludhiana in panels 2014–2021.

**FIGURE 6:**
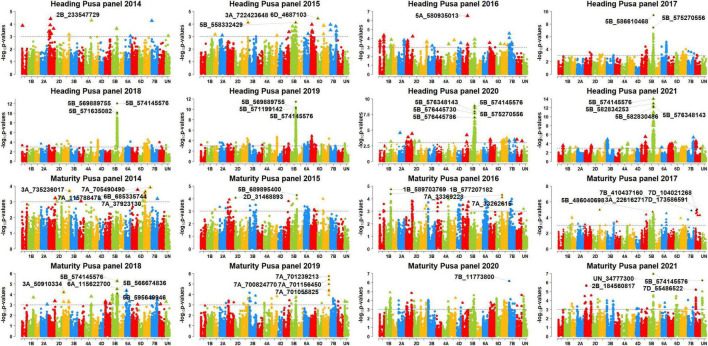
Manhattan plots showing the genomic regions significantly associated with days to heading (DH) and maturity (DM) in Pusa in panels 2014–2021.

We observed that three markers (5B_562646635, 5B_569889755, and 5B_574145576) were significantly associated with DH in all the three sites (Figures 4–6) and they explained 4.3–4.7, 4.4–9.7, and 5.2–14.07% of the phenotypic variation in different sites, respectively. In addition, marker 5A_580935013 and 72 markers on chromosome 5B between 549730988 and 597098833 bps were associated with DH in Jabalpur and Pusa. These markers had additive effects ranging between 1 and 2.8 days in Jabalpur and between 1 and 4.4 days in Pusa and explained 4.3–10.3 and 4.3–13% of the phenotypic variation in Jabalpur and Pusa, respectively. Three markers on chromosome 7D including 7D_55920290, 7D_57904794, and 7D_58369927 were significantly associated with DH in Jabalpur and Ludhiana. These markers had additive effects ranging between 0.94 and 0.99 days in Jabalpur and 0.87 and 0.96 days in Ludhiana, while they explained 4.8–6.6 and 4.9–6.2% of the phenotypic variation in Jabalpur and Ludhiana, respectively. Quantile–quantile plots demonstrating the ratios of expected to observe log10 (*P*) values for DH and DM at each location are presented in Supplementary File 5.

### Markers Significantly Associated With Days to Maturity in More Than One Site

We observed that markers 5B_574145576 and 7D_55486522 were associated with DM in Jabalpur and Pusa. Among them, marker 5B_574145576 had additive effects ranging from 1.2 to 1.8 days in Jabalpur and 0.95 to 1.1 days in Pusa and explained 4.6 to 9.5% and 4.7 to 6.4% of the phenotypic variation in Jabalpur and Pusa, respectively. The other marker (7D_55486522) had additive effects of 1.1 and 0.66 days in Jabalpur and Pusa, respectively, and it explained 4.8 and 5% of the phenotypic variation in these sites. Furthermore, three markers on 7D (7D_55529933, 7D_55920290, and 7D_57904794) were found to be significantly associated in Jabalpur and Ludhiana. These markers had additive effects ranging from 1.06 to 1.1 days in Jabalpur and 0.67 to 0.68 days in Ludhiana, while they explained 4.7 to 5.2% and 4.7 to 5.5% of the phenotypic variation in Jabalpur and Ludhiana, respectively.

### Markers Significantly Associated With Both Days to Heading and Days to Maturity

Eighty-four markers were significantly associated with both traits DH and DM in different sites and panels (Figures 4–6) including 41 markers on chromosome 5B (between 557138254 and 595649946 bps), 18 markers on chromosome 2B (between 43232877 and 59584538 bps), 10 markers on chromosome 7D (between 55446953, and 58633321 bps), eight markers on chromosome 4D (between 108499424 and 368596770 bps), four markers on chromosome 6B (between 186319977 and 224585504 bps), and one marker each on chromosomes 3D, 5A, and 7B.

## Discussion

We have analyzed the variation in DH and DM in three main wheat-producing zones in India, including Ludhiana, representing the NWPZ, Jabalpur, representing the CZ and Pusa, representing the NEPZ. We observed that across all the panels, the mean DH was the highest in Ludhiana (103.8 ± 6 days) and lowest in Jabalpur (77.7 ± 6.7 days), while the mean DM were the highest in Ludhiana (148.6 ± 4.6 days) and lowest in Jabalpur (121.6 ± 4.9). This could be because of local weather conditions, mainly temperature which remains cooler in the northwest India where Punjab (Ludhiana) is located compared with central India which has the state of Madhya Pradesh (Jabalpur). Among the three sites, the mean broad-sense heritabilities for DH were higher in Ludhiana (0.93 ± 0.03) and Jabalpur (0.84 ± 0.09) than in Pusa (0.85 ± 0.07). The high heritabilities that we have observed, along with the high genetic variance compared with the residual variance in all the sites, indicate the high contribution of genetic factors to the phenological traits in the panels and environments analyzed in this study. Our analysis of variance results across sites indicated that both the genotypes and sites were significantly different for both the phenological traits, which indicates the variable performance of the genotypes in the different sites and also the environmental differences in these sites. Our results also indicated that among the three sites, the highest phenotypic correlation for DH (0.69 ± 0.15) and DM (0.61 ± 0.08) was between Pusa and Jabalpur, while the lower correlation was between Pusa and Ludhiana (0.59 ± 0.09, 0.52 ± 0.13) for DH and DM, respectively. This could be due to the fact that wheat-growing seasons at Pusa and Jabalpur are warmer and about 2–3 weeks shorter than Ludhiana. The shorter crop duration at Pusa and Jabalpur had effect on phenological traits such as DH and DM. However, Pusa is more humid compared with Jabalpur and Ludhiana.

We performed GWAS for DH and DM and reported 222 markers significantly associated with the traits in different spring bread wheat panels. On chromosome 2BL, several markers between 43232877 and 59584538 bps were significantly associated with both heading and maturity in Ludhiana with additive effects ranging between 0.62 and 2.1 days. Among them, marker 2B_56134146 that was significant in seven datasets was the closest marker to the *Ppd*-B1 gene (0.10 Mbs away) and is therefore linked to the gene. However, we also observed that the *Ppd*-B1 gene was associated with the phenological traits in only Ludhiana representing the NWPZ and not the other sites, indicating that it contributes to specific adaptation to this zone. Díaz et al. (2012) showed that alleles of *Ppd*-B1 (along with *Vrn*-A1) were associated with an increased copy number of both genes and resulted in earlier flowering (*Ppd*-B1a) or increased vernalization requirements (*Vrn*-A1w). These results, along with a separate study in durum (Würschum et al., 2019), suggest that copy number variation is important for the adaptation of wheat.

On chromosome 4D, we have identified markers between 61853654 and 368596770 bps associated with both DH and DM in Ludhiana with additive effects ranging between 0.2 and 1.6 days. Among them, marker 4D_368596770 was very close to the *Vrn*-D2 gene (within 141 kb) and appears to indicate a novel locus. Marker 5A_580935013 on 5AL in panel 2016 was associated with DH in Jabalpur and Pusa and DM in Jabalpur. This marker had the additive effects ranging between 2 and 4.4 days and is linked to the *Vrn*-A1 gene which is only 6.5 Mbps away. However, possibility of some other genes in that region may not be ruled out. Our results indicate that this gene does not play a major role in the phenology and adaptation to the three major wheat-producing zones of India in the lines and environments used in this study. The *Vrn*-A1 gene, which is known to play a role in frost tolerance, did not appear important as tested in this study, but has shown an increased frequency of its alleles in winter wheat (Eagles et al., 2011; Zhu et al., 2014). A previous study by Juliana et al. (2019) also did not observe a significant effect of the *Vrn*-A1 gene on phenology in CIMMYT’s advanced spring wheat breeding lines. These results are in contrast to those reported by Santra et al. (2009), which indicated *Vrn*-A1a as the most frequent allele in spring wheat genotypes from the Pacific Northwest region of the United States.

On chromosome 5BL, several markers between 549730988 and 597098833 were associated with DH in six panels in Jabalpur, two in Ludhiana, and five in Pusa. In addition, they were also associated with DM in three panels in Jabalpur and two in Pusa. While the additive effects of the 5B markers ranged between 0.8 and 2.8 days, the marker 5B_574145576 that was significantly associated in 16 datasets was only 0.33 Mbs away from the *Vrn*-B1 gene. However, we observed that the *Vrn*-B1 gene was associated with DH in multiple panels in Jabalpur and Pusa, but only in a few panels in Ludhiana, indicating its strong association with phenology in multiple years at the NEPZ and CZ, compared with the NWPZ of India.

On chromosome 6B, markers between 186319977 and 224585504 bps were associated with both heading and maturity in Jabalpur in panel 2015 and suggest a specific adaptation QTL for Jabalpur, with additive effects ranging between 1 and 1.1 days. These markers flank *Qcim.6B.5.2* linked to marker S6B_190376868 that was associated with DH in irrigated and drought-stressed environments of Obregon, Mexico (Juliana et al., 2019), and indicate the presence of the same QTL. On chromosome 7BS, markers 7B_8219840 and 7B_11773800 that were associated with DM in Jabalpur and Pusa with additive effects ranging between 0.9 and 1 days flanked the *Vrn*-B3 gene but were significant only in panel 2020. On chromosome 7DS, several markers between 55446953 and 58633321 bps were associated with DH and DM in panels 2020 and 2021 in Ludhiana and Jabalpur and DM in only panel 2021 in Pusa and had additive effects ranging between 0.6 and 1.1 days. While marker 7D_58633321 was 9.8 Kbp away from the *Vrn*-D3 gene and might be indicating this gene, the markers were in the same position as *Qcim.7D.2* associated with DM in irrigated and drought-stressed environments in Obregon, Mexico (Juliana et al., 2019).

While this study has validated genomic regions associated with DH and maturity in previous studies, we have also reported novel genomic regions not reported previously. The presence of *Ppd*-B1 genes in all the well-adapted lines of each panel suggests the importance of those genes in NWPZ, while the *Vrn*-B1 gene is indicated to be contributed to adaptation in the NEPZ and CZ of India. We observed a maximum variation of 21.1 and 14% explained by markers 2B_56134146 and 5B_574145576 associated with the *Ppd*-B1 and *Vrn*-B1 genes’ regions, respectively, that further provide evidence to their significant role in regulating DH and maturity. We also observed that 9.1–77.3% of the markers significantly associated with DH and 2–9% of the markers significantly associated with DM were significantly associated in more than one panel, indicating a large effect of the years and environments on the traits. In addition, this could also be attributed to different lines with variable allelic frequencies that were present in the different panels and because not all the markers used for GWAS were common across all the panels.

The average extent of LD in wheat was approximately 5 × 10^7^ bps (Juliana et al., 2018). Therefore, finding association of candidate genes with significant markers is difficult due to the high LD in many chromosomal regions/gene intervals. Extensive LD in wheat that decays at about 5 × 10^7^ bps poses a huge challenge for delineating candidate gene intervals, and candidates should be further fine mapped, functionally characterized, and validated (Juliana et al., 2018).

**FIGURE 7:**
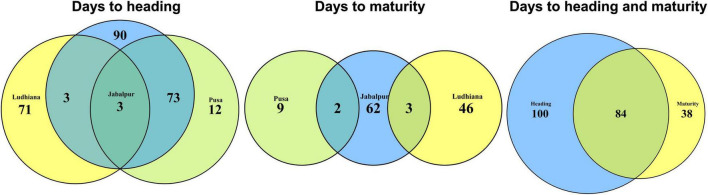
Venn diagrams showing the markers significantly associated with days to heading (DH) and maturity (DM) in different sites and the overlapping number of markers across sites and traits.

The GWAS results for DH indicated that three markers on chromosome 5B (in proximity of Vrn-B1 gene) were constantly significant in all environments, while 72 markers were significant in Jabalpur and Pusa. Similarly, three markers on chromosome 7D were significant in Jabalpur and Ludhiana. This indicates the existence of shared genetic basis for this trait and adaption in different wheat-producing zones of India. However, for DM we observed that no marker was associated with all the three sites, while two to three markers were common between Jabalpur and Pusa, as well as Jabalpur and Ludhiana (Figure 7). While this indicates that there could be a shared genetic basis for DM in these sites, it is also worth highlighting that none of the markers were significantly associated with DM in both Ludhiana and Pusa. Finally, we also observed 84 markers that were associated with both DH and maturity in the different panels and sites with most of them linked to the *Ppd*-B1 and *Vrn*-B1 genes indicating the association of these genes with both traits. Adjusting the developmental stages and maturity of wheat through breeding is one of the best ways to develop widely adapted varieties. The combination of *Vrn* and *Ppd* genes plays an important role in adaptation to a particular environment. For example, higher productivity is obtained under early sown wheat where mild vernalization gene and relatively significant effect *Ppd* gene combination is preferred (Farhad et al., 2022). The results of this study are expected to provide valuable insights into the genetic basis of phenology-driven adaptation of bread wheat genotypes in the major wheat-producing zones of India.

## Data Availability Statement

The original contributions presented in this study are included in the article/Supplementary Material, further inquiries can be directed to the corresponding authors.

## Author Contributions

PKB wrote the manuscript. PKB and PJ analyzed the data and wrote, reviewed, and edited. RPS, JH-E, LC-H, VG, and SM provided the breeding material and review and edited. JP and SS provided genotyping data. PKB, MKV, and UK managed field trials and recorded the phenotypic data. UK, RPS, AKJ, and SM designed the experiment and supervised the research project conceptualization, funding acquisition, project administration, resources, supervision, review, and editing. All authors have read and approved the final manuscript.

## Conflict of Interest

The authors declare that the research was conducted in the absence of any commercial or financial relationships that could be construed as a potential conflict of interest.

## Publisher’s Note

All claims expressed in this article are solely those of the authors and do not necessarily represent those of their affiliated organizations, or those of the publisher, the editors and the reviewers. Any product that may be evaluated in this article, or claim that may be made by its manufacturer, is not guaranteed or endorsed by the publisher.

## Footnotes

1
https://eands.dacnet.nic.in/

2
https://iiwbr.icar.gov.in/

3
http://visualization.ritchielab.org/phenograms/plot

## Supplementary Material

Supplementary File 1 Biplot and dendrogram of the genetic correlation matrix for the traits studied in the wheat association panel grown in three different environments in India from 2014–2021.

Supplementary File 2 Individual marker effect, position on each chromosome, and *p*-value with marker effect.

Supplementary File 3 Single-nucleotide polymorphism markers significantly associated with phenological traits (DH and DM).

Supplementary File 4 Consistent markers significantly associated with agro-morphological traits.

Supplementary File 5 Quantile–quantile plots demonstrating the ratios of expected to observe log10 (*P*) values.
