# Supplementary material for: Dissecting the Genetic Architecture of Phenology Affecting Adaptation of Spring Bread Wheat Genotypes to the Major Wheat-Producing Zones in India
Source: Front Plant Sci. 2022 Jul 6;13:920682. doi: 10.3389/fpls.2022.920682 (PMC9298574; doi:10.3389/fpls.2022.920682)

**Supplementary File: 1** Bi-plot and dendrogram of the genetic correlation matrix for the traits studied in the wheat association panel grown in three different environments in India from 2014 to 2021

**2013-14**


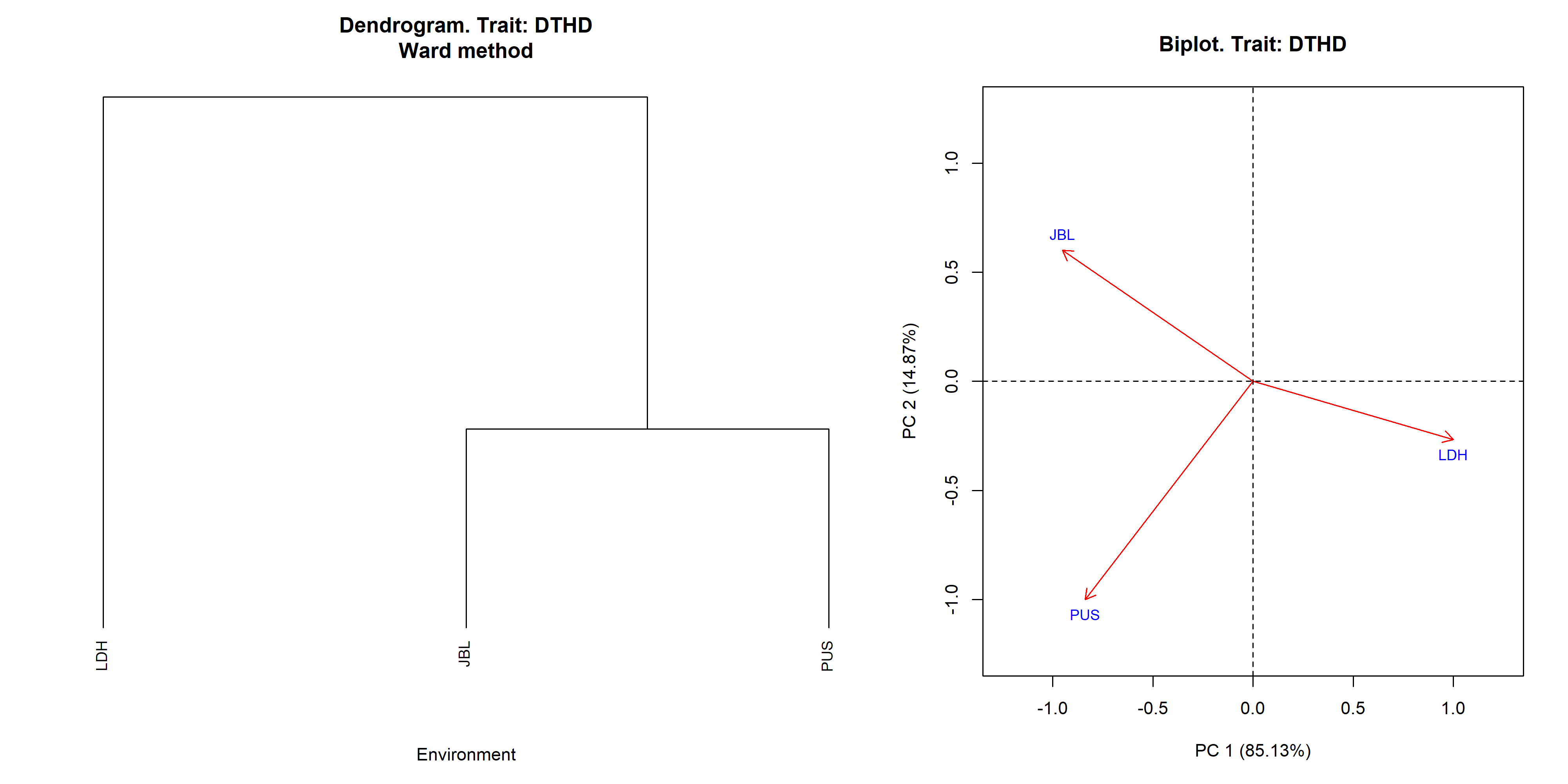


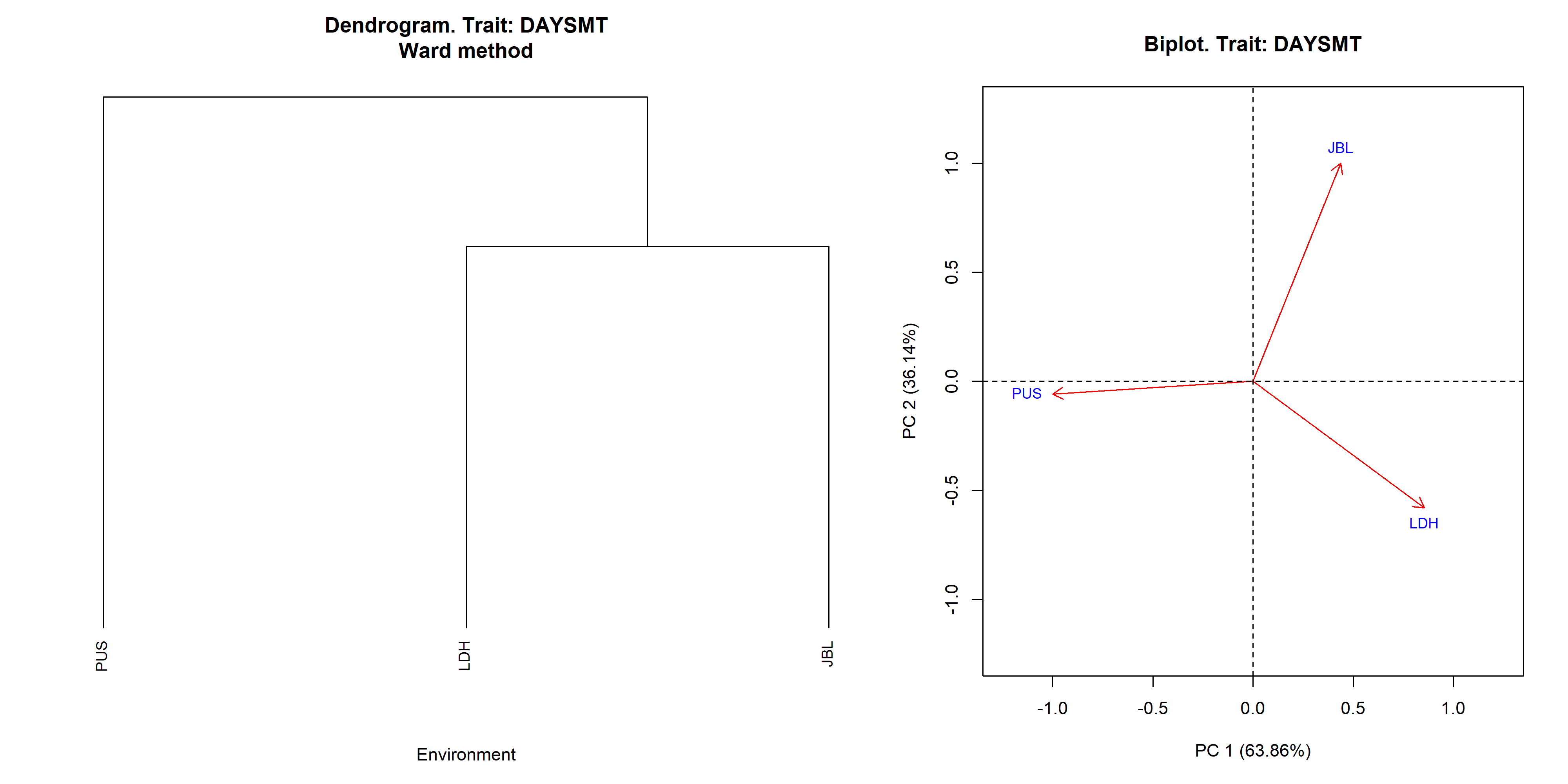


**2014-15**
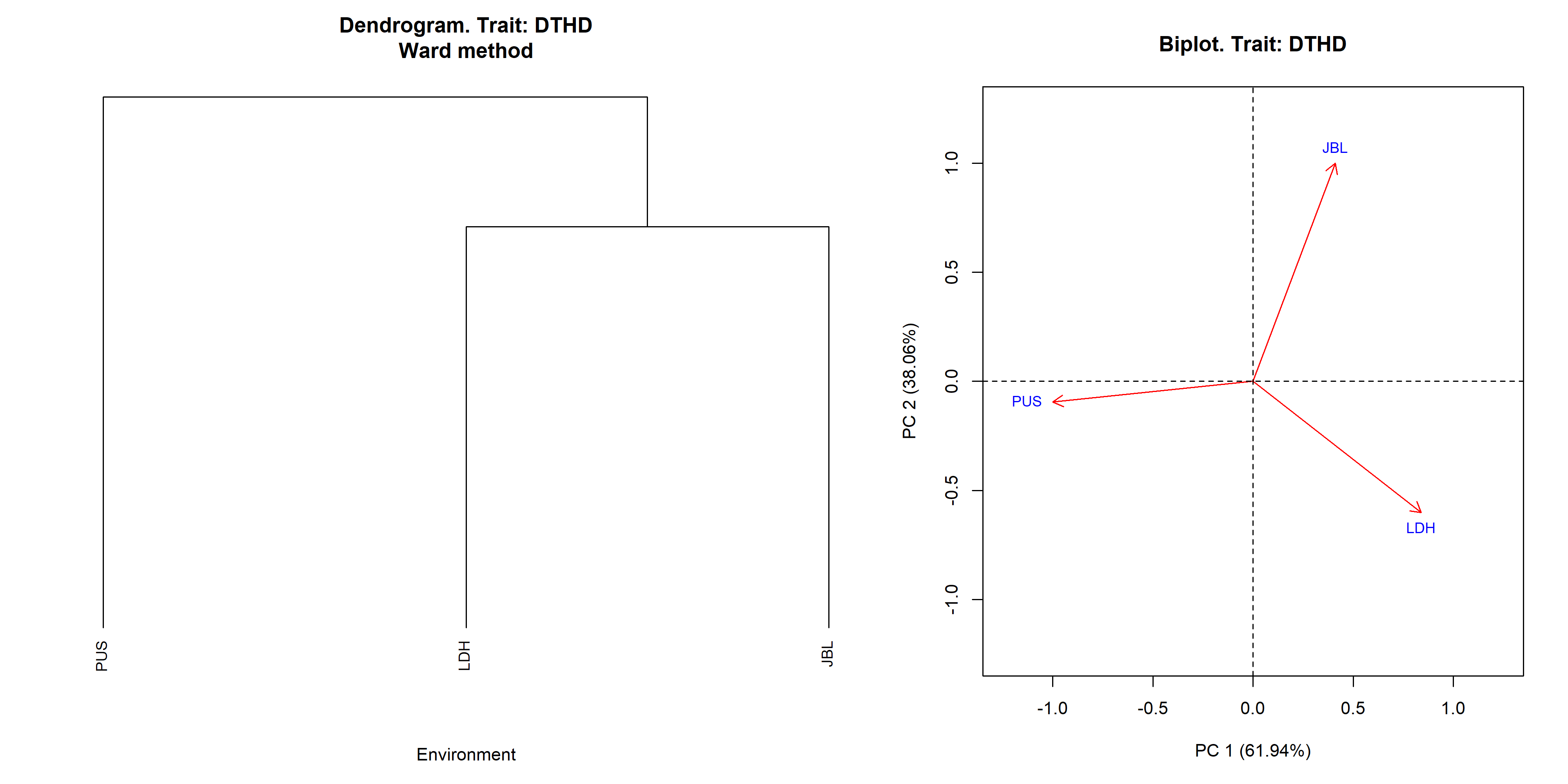


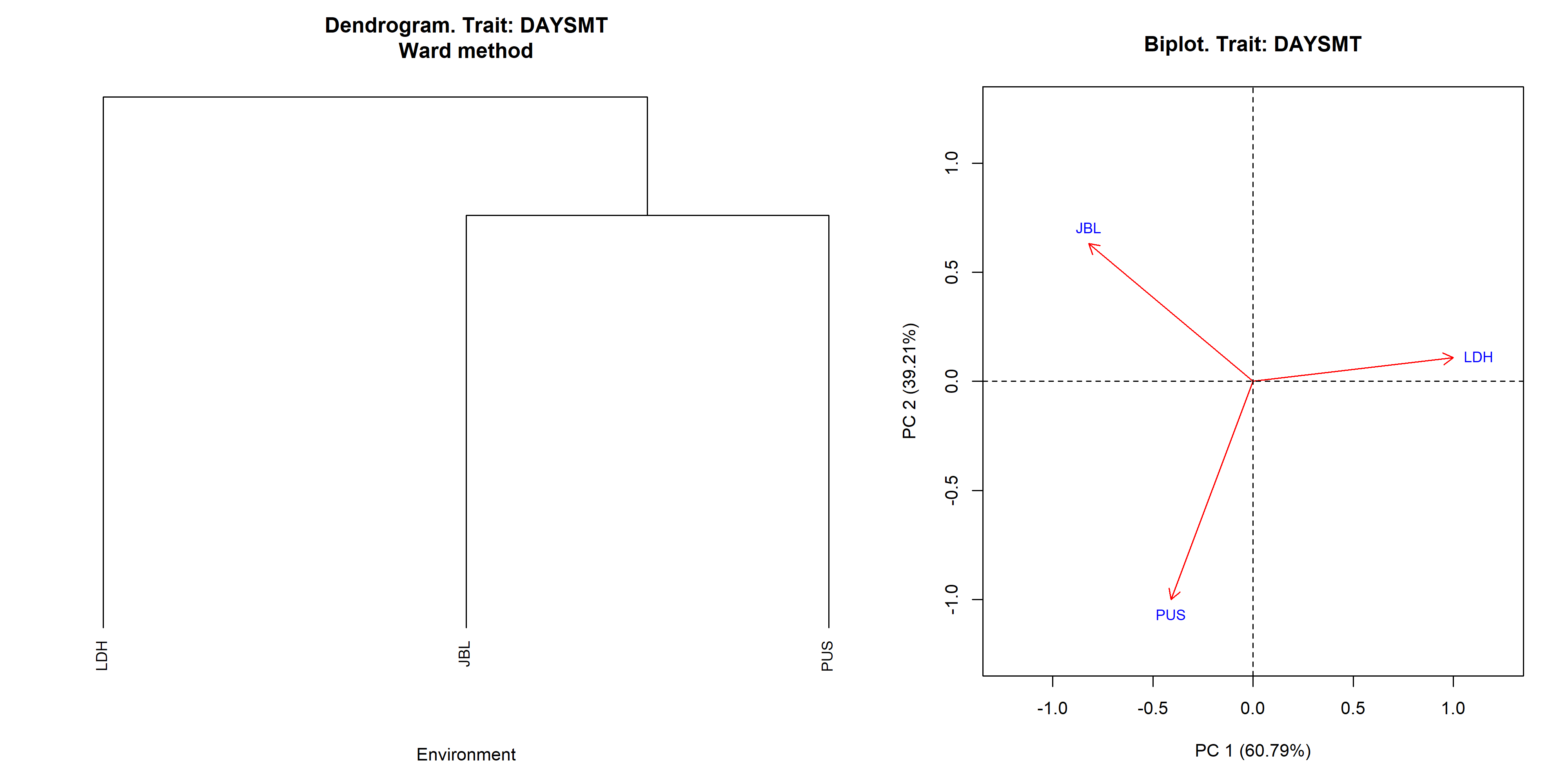


**2015-16**


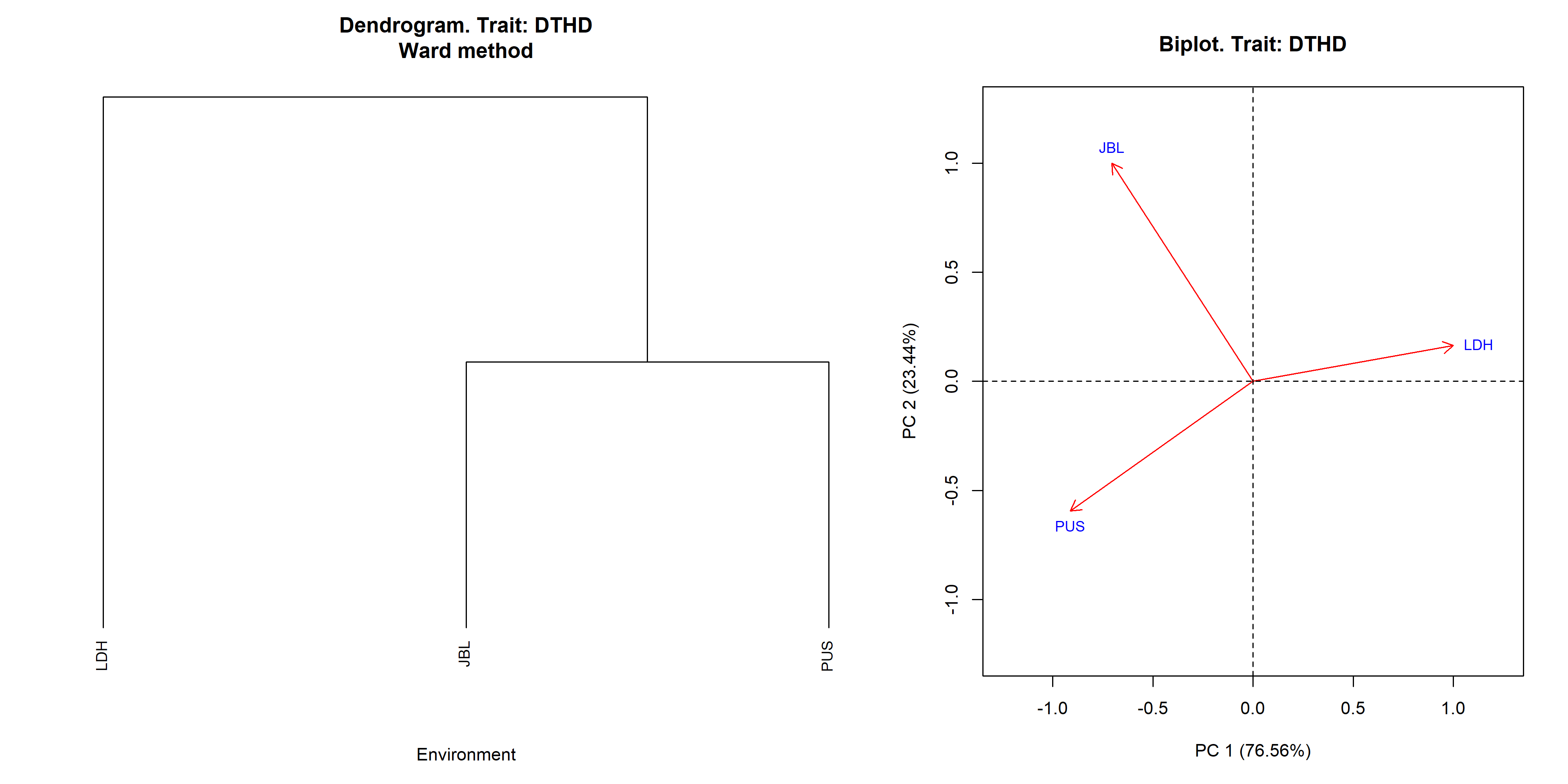


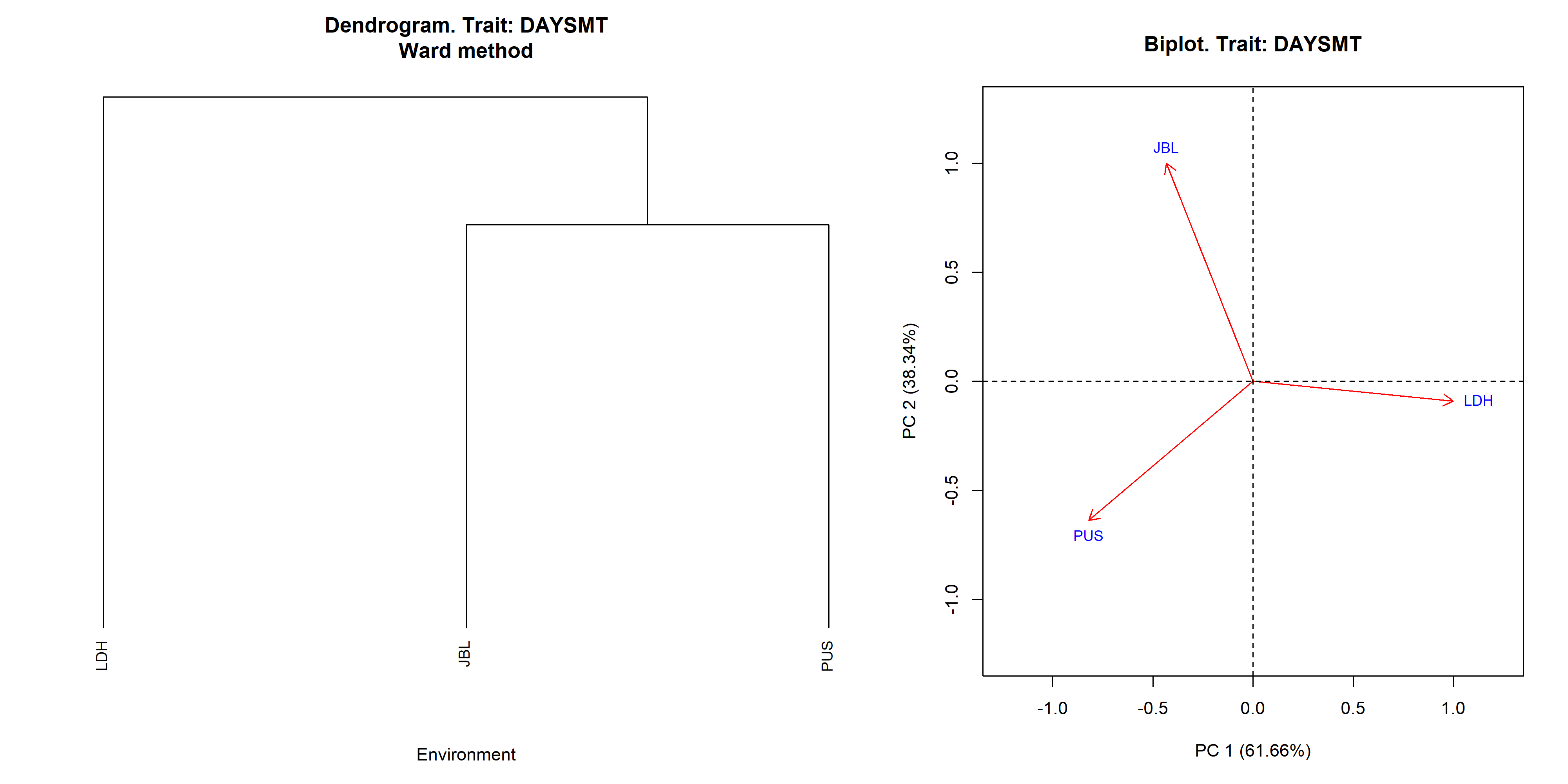


**2016-17**


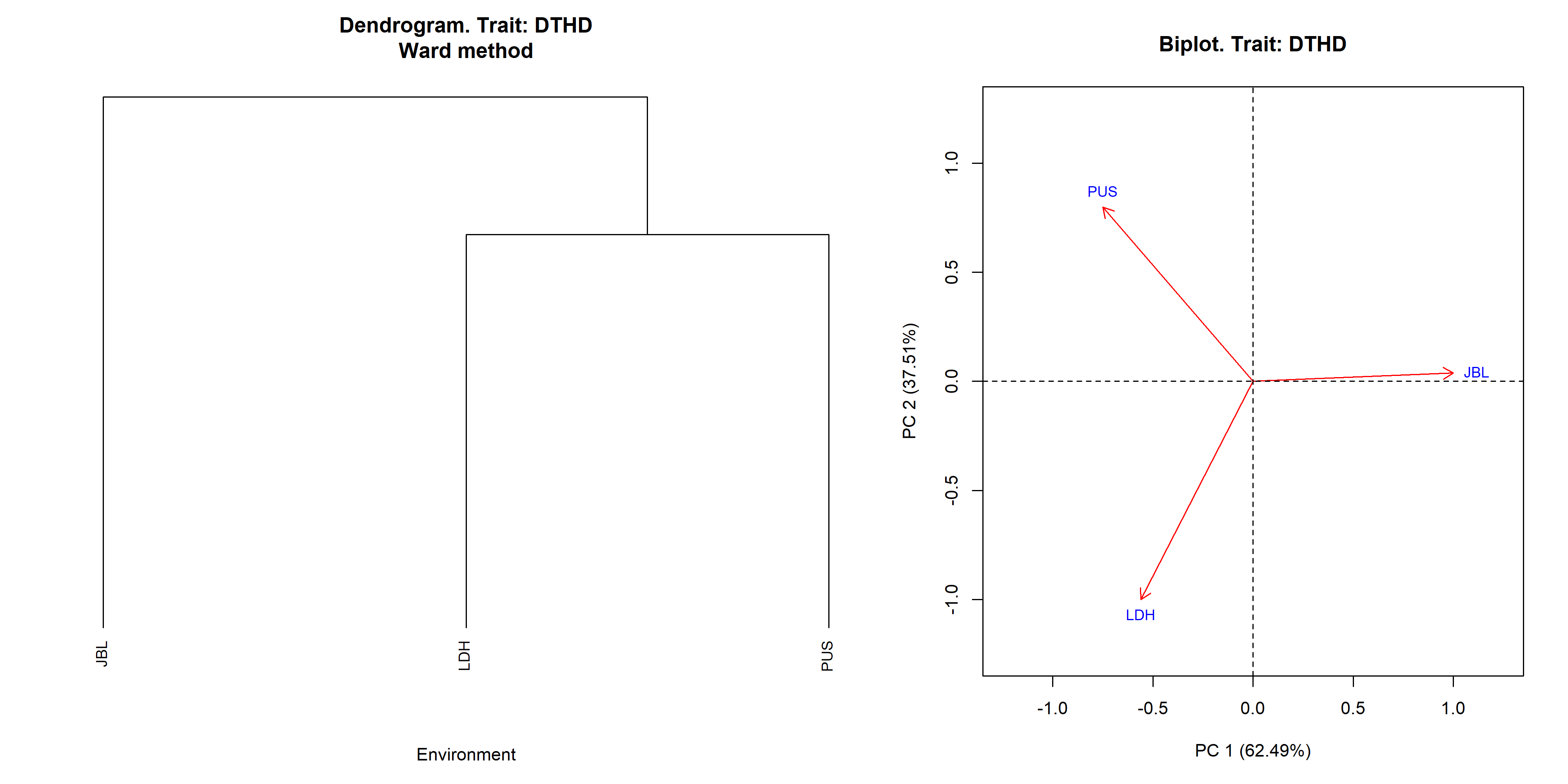


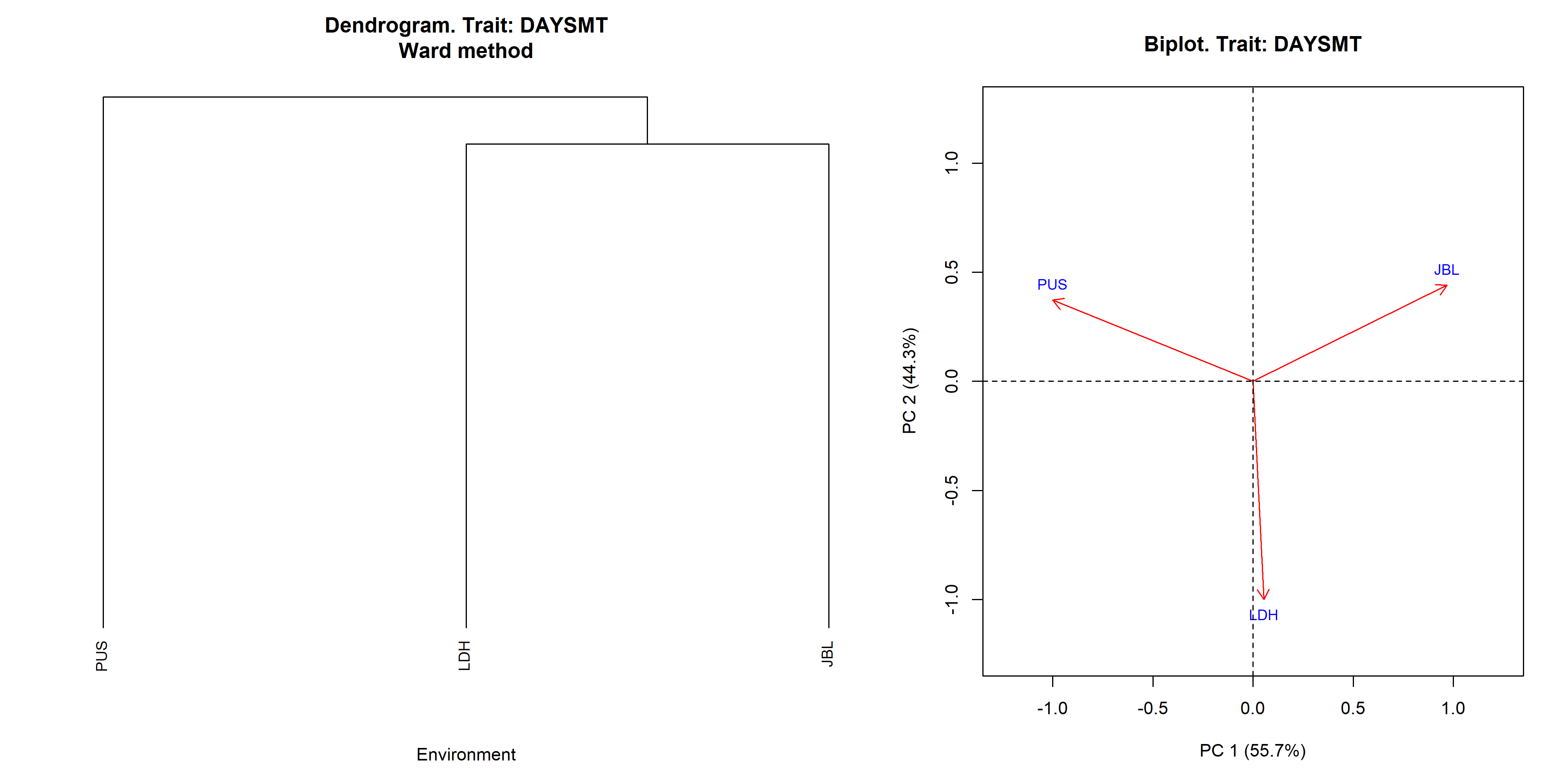


**2017-18**


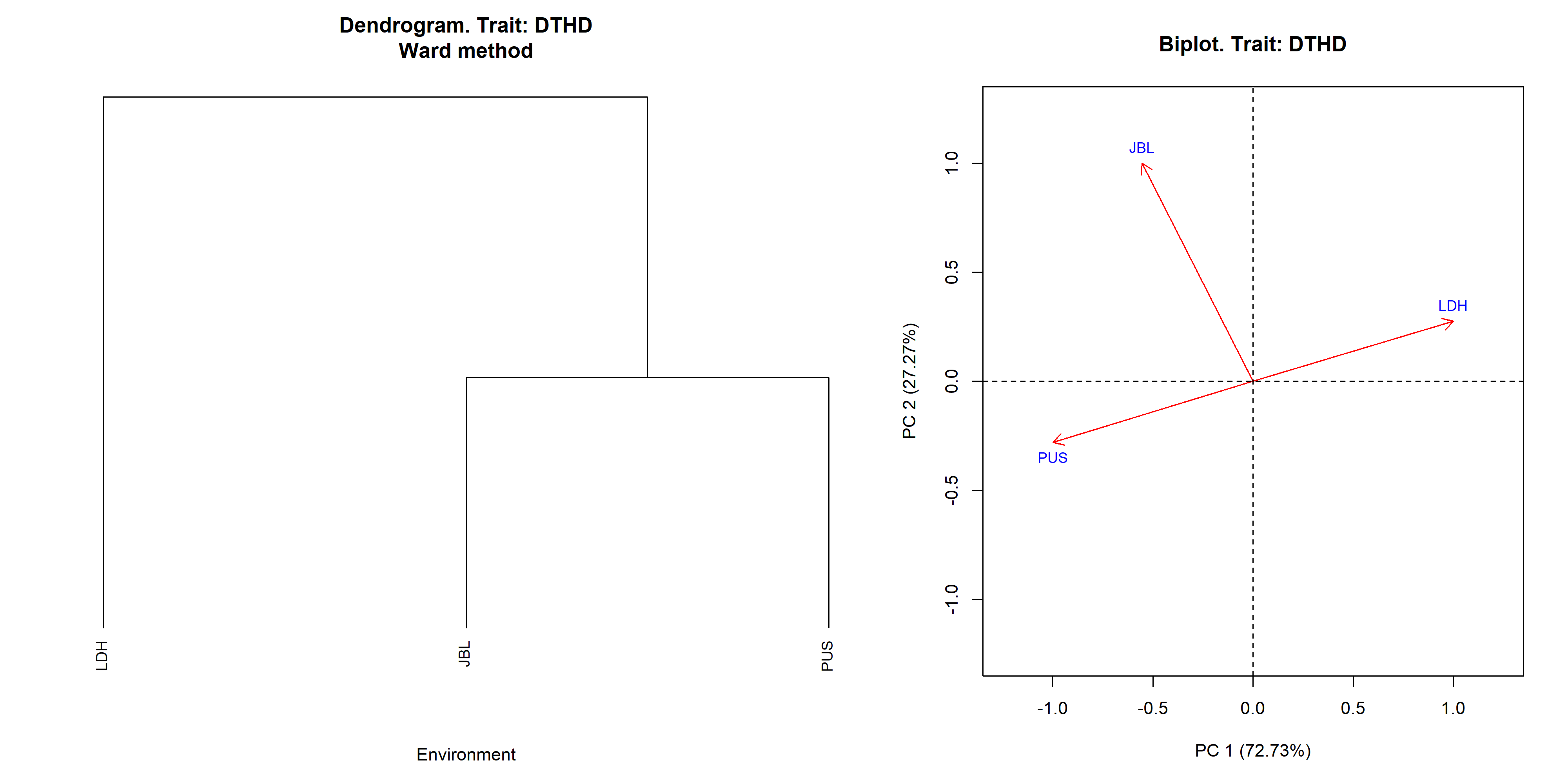


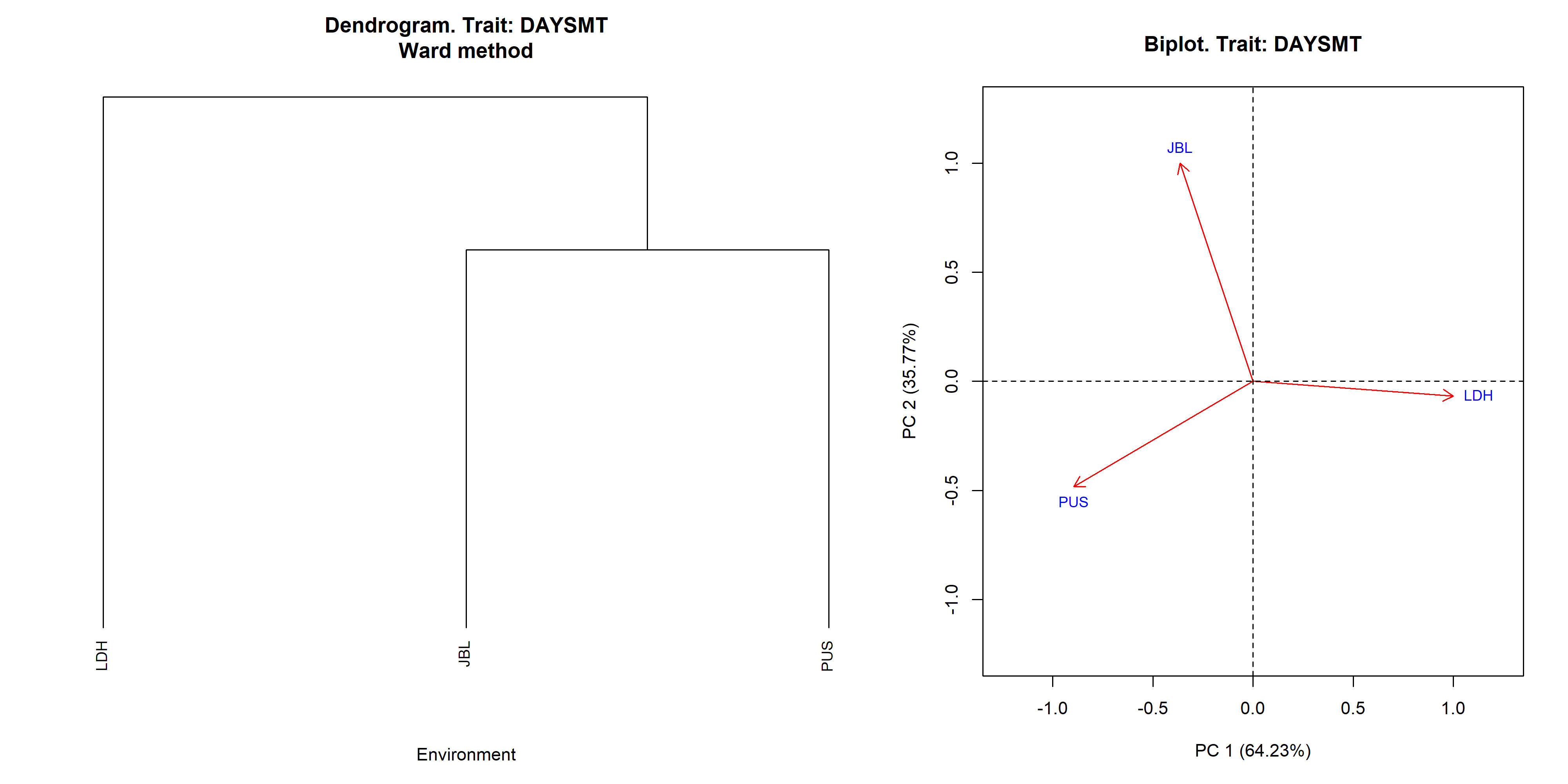


**2018-19**


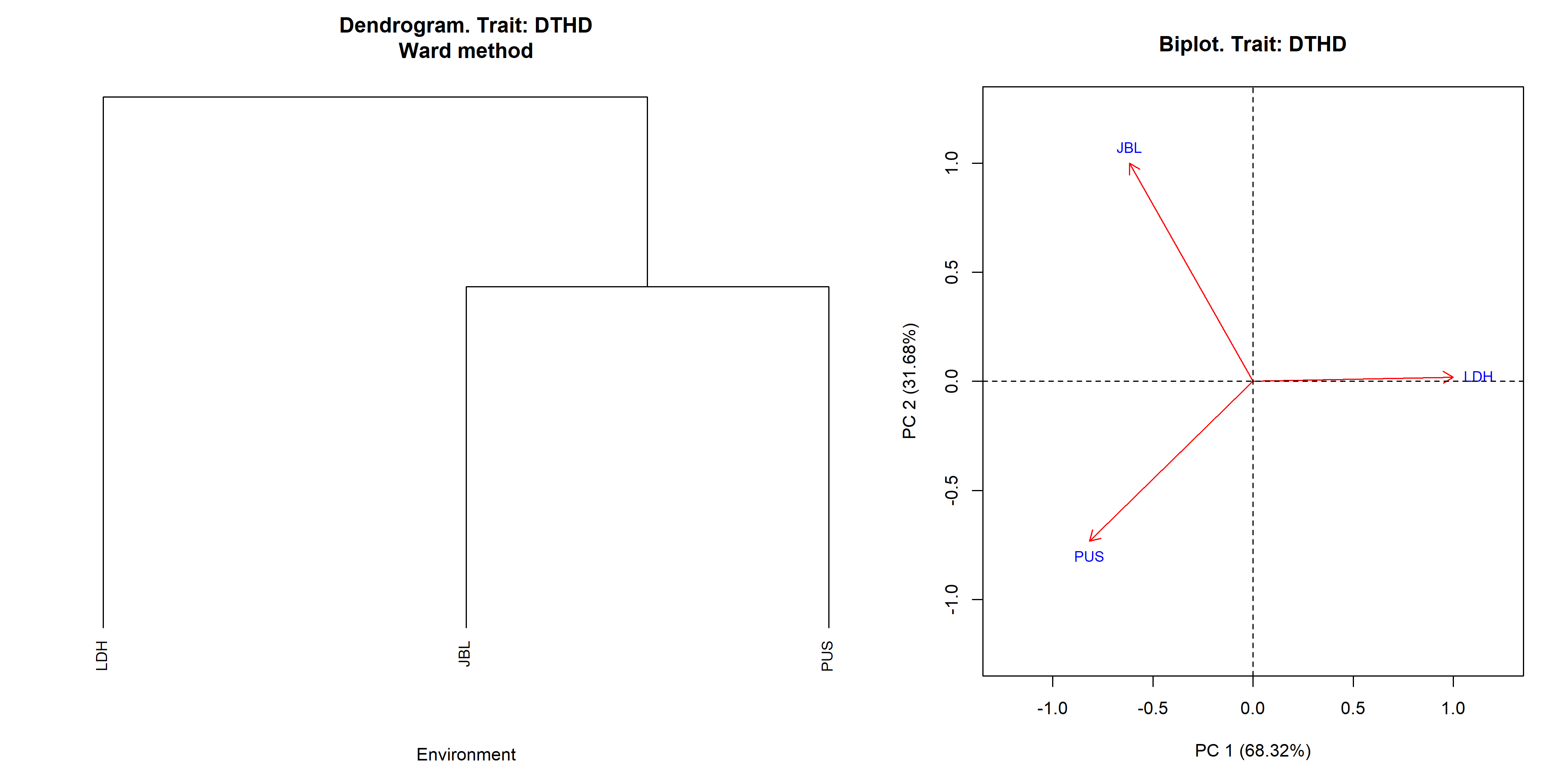

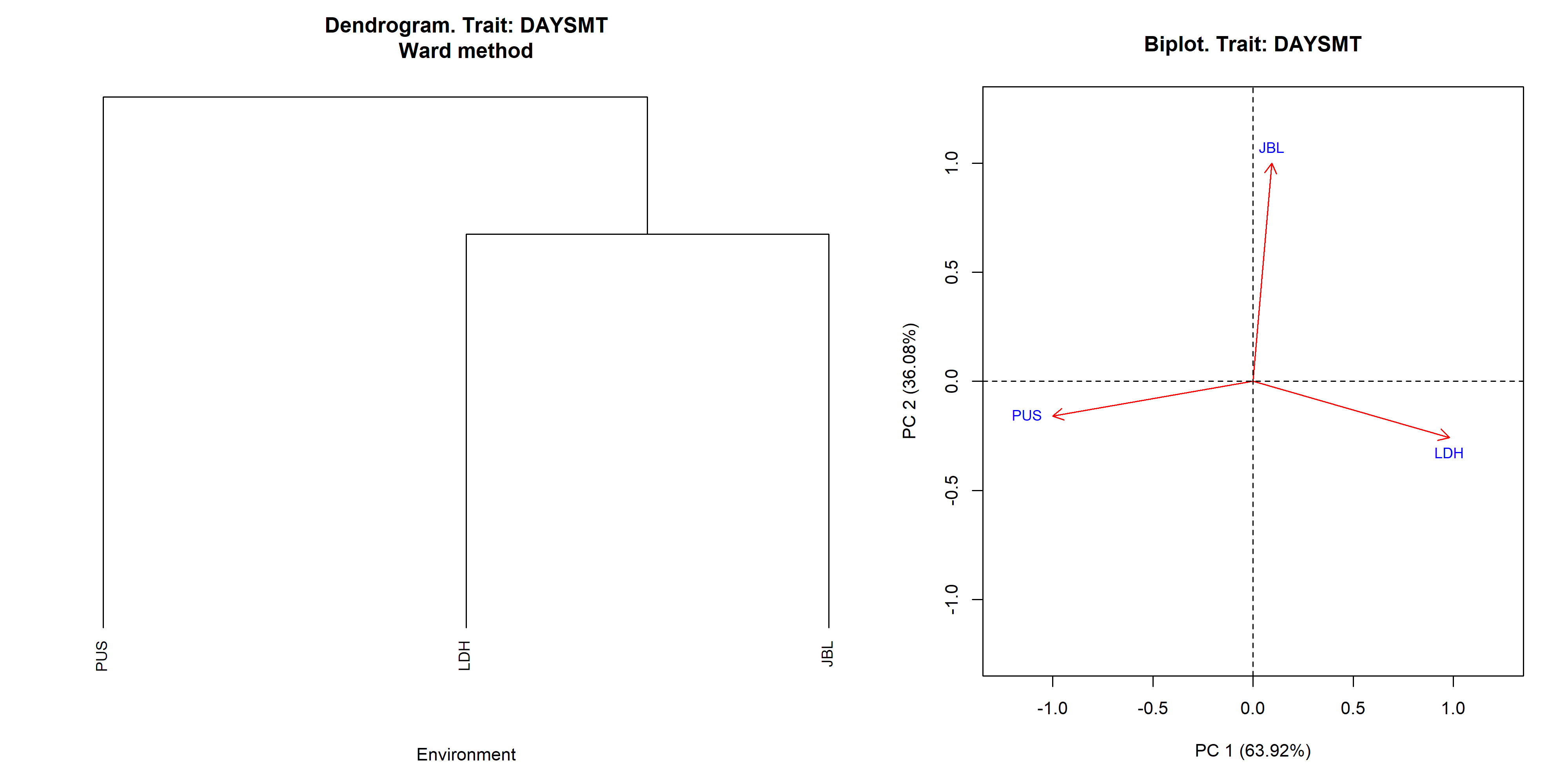


**2019-20**


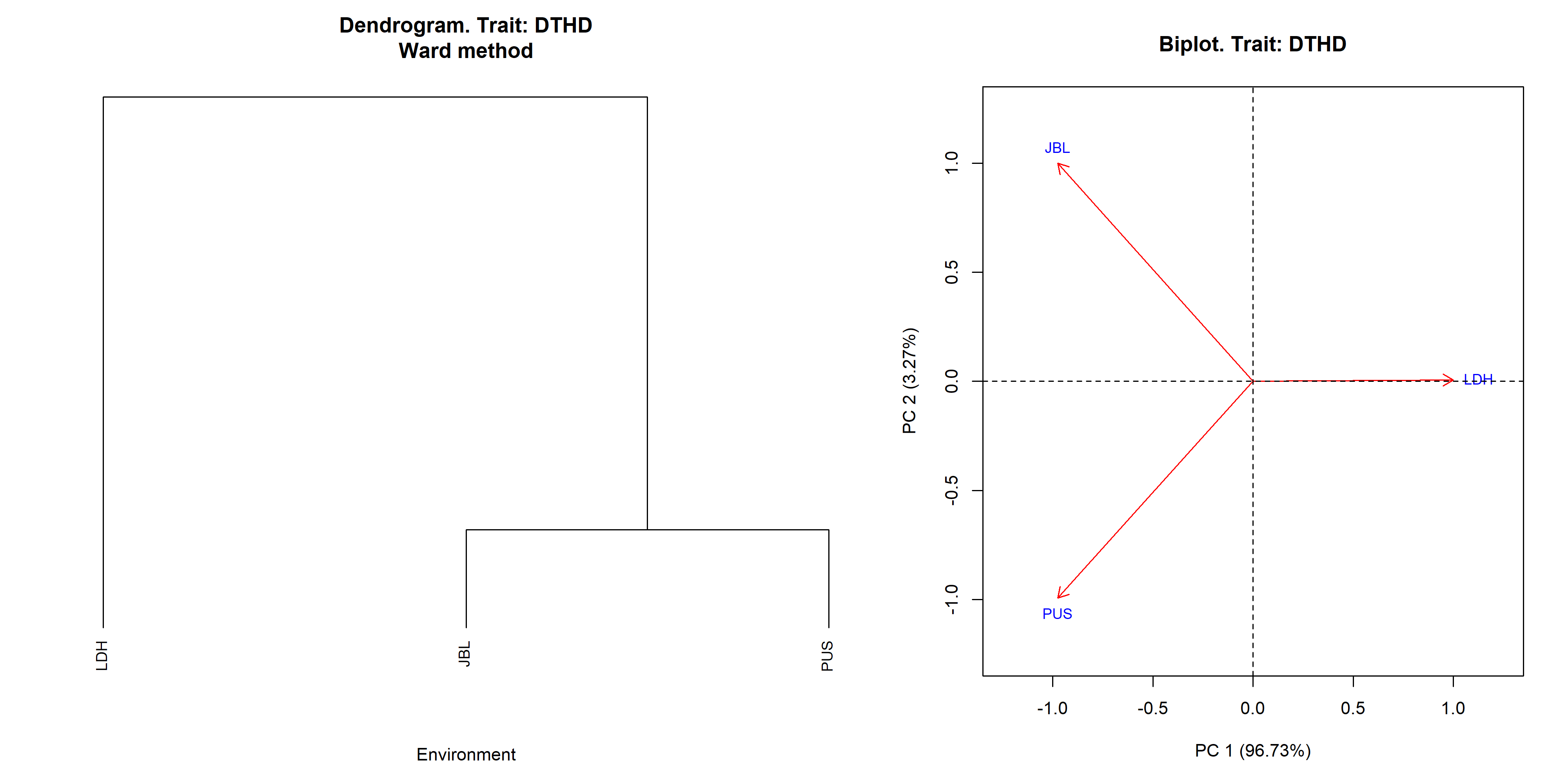


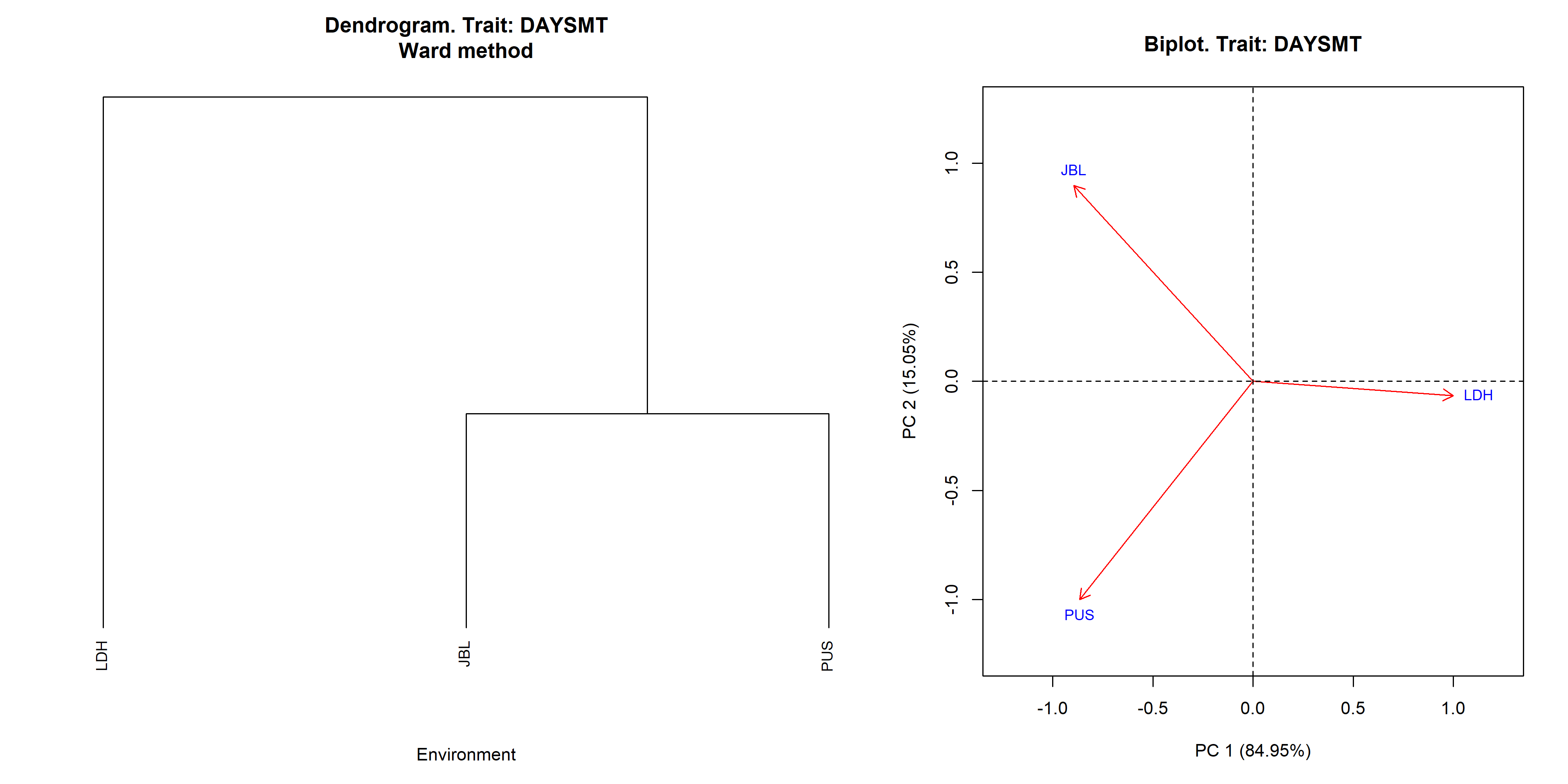


**2020-21**
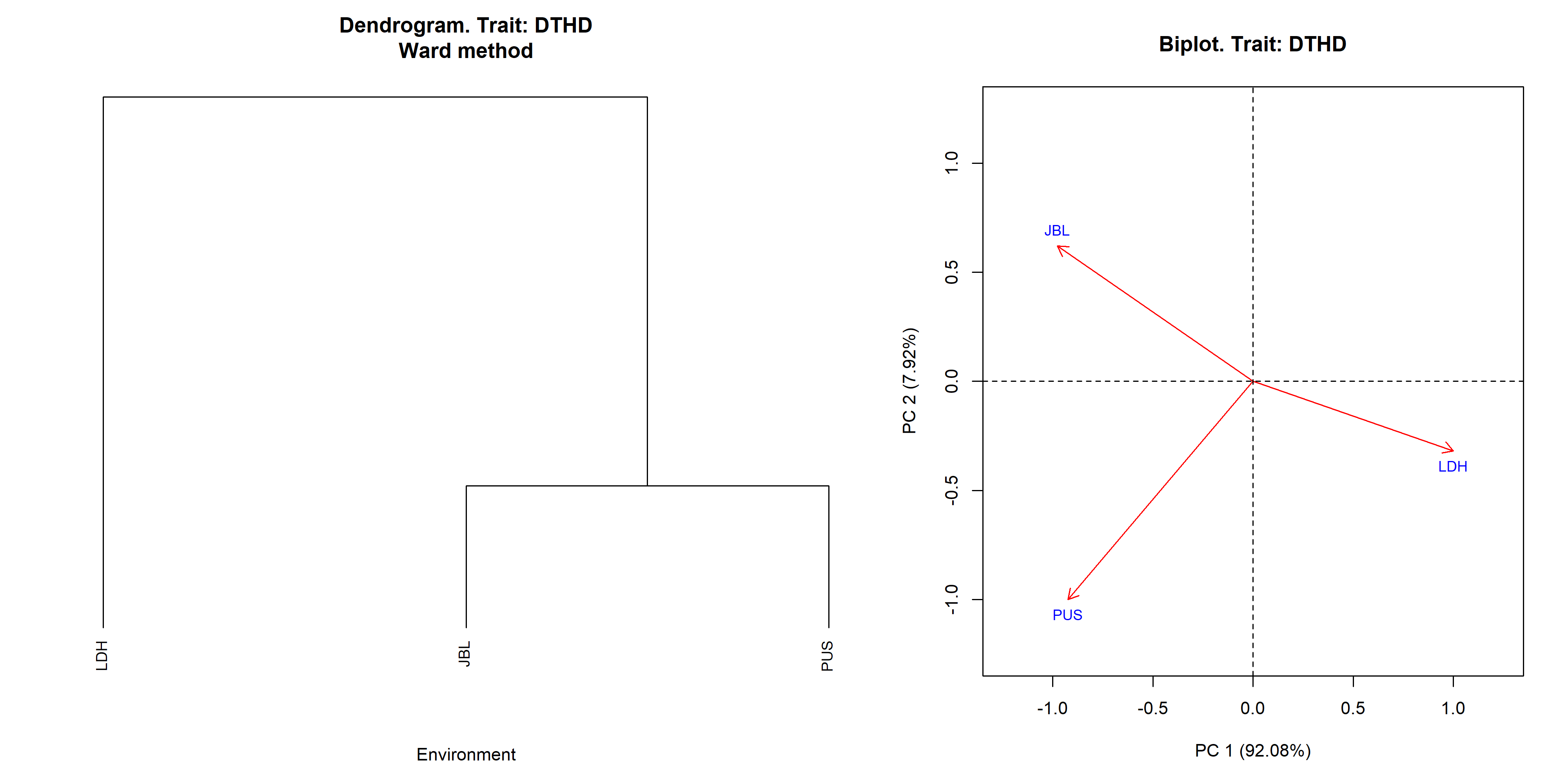

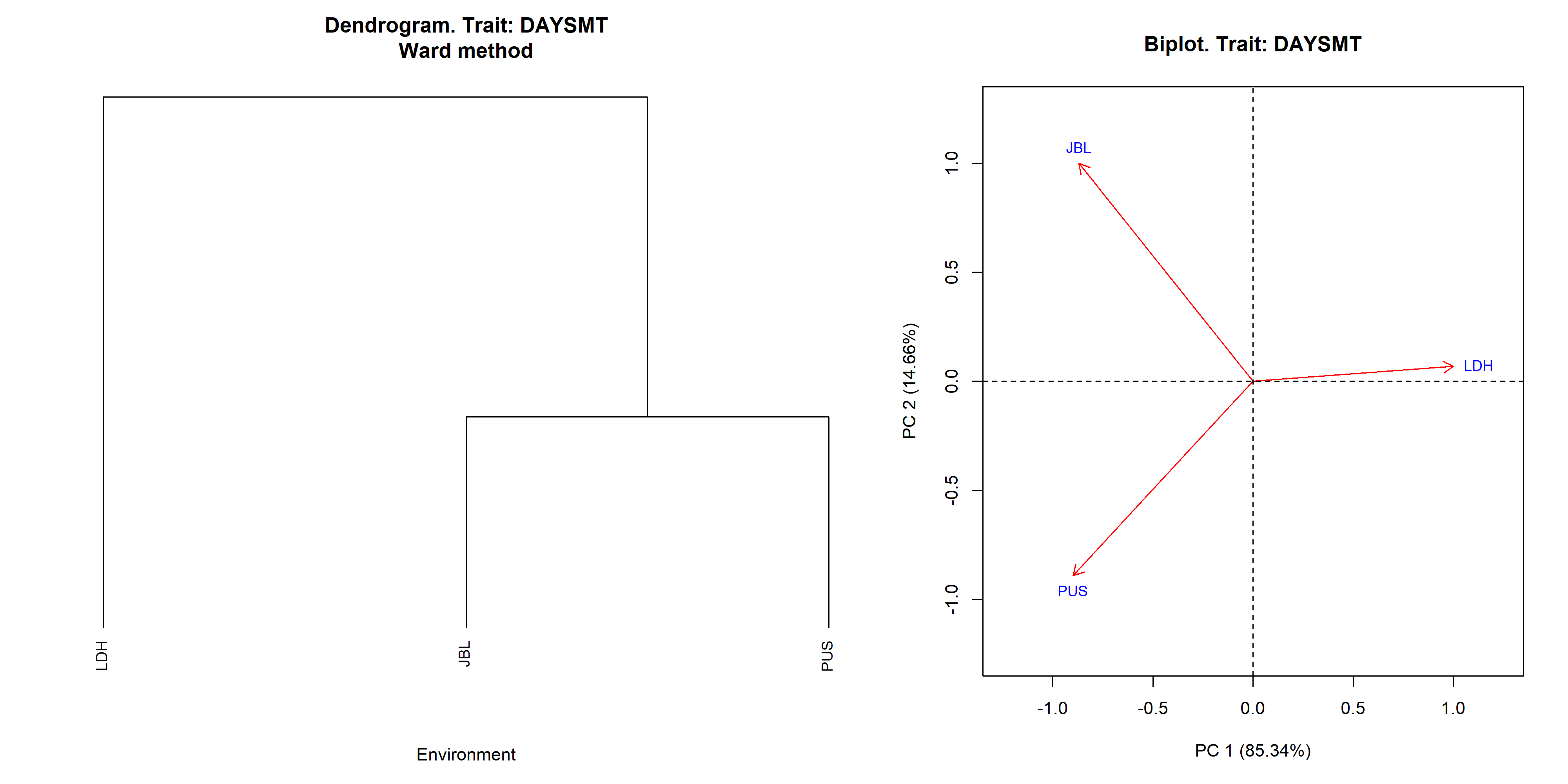

Supplement: Supplementary File 1 — Biplot and dendrogram of the genetic correlation matrix for the traits studied in the wheat association panel grown in three different environments in India from 2014–2021. [file Table_1.DOCX]
