# Supplementary material for: Dissecting the Genetic Architecture of Phenology Affecting Adaptation of Spring Bread Wheat Genotypes to the Major Wheat-Producing Zones in India
Source: Front Plant Sci. 2022 Jul 6;13:920682. doi: 10.3389/fpls.2022.920682 (PMC9298574; doi:10.3389/fpls.2022.920682)
Supplement: Supplementary File 5 — Quantile–quantile plots demonstrating the ratios of expected to observe log10 (P) values. [file Presentation_1.PPTX]

## Slide 1
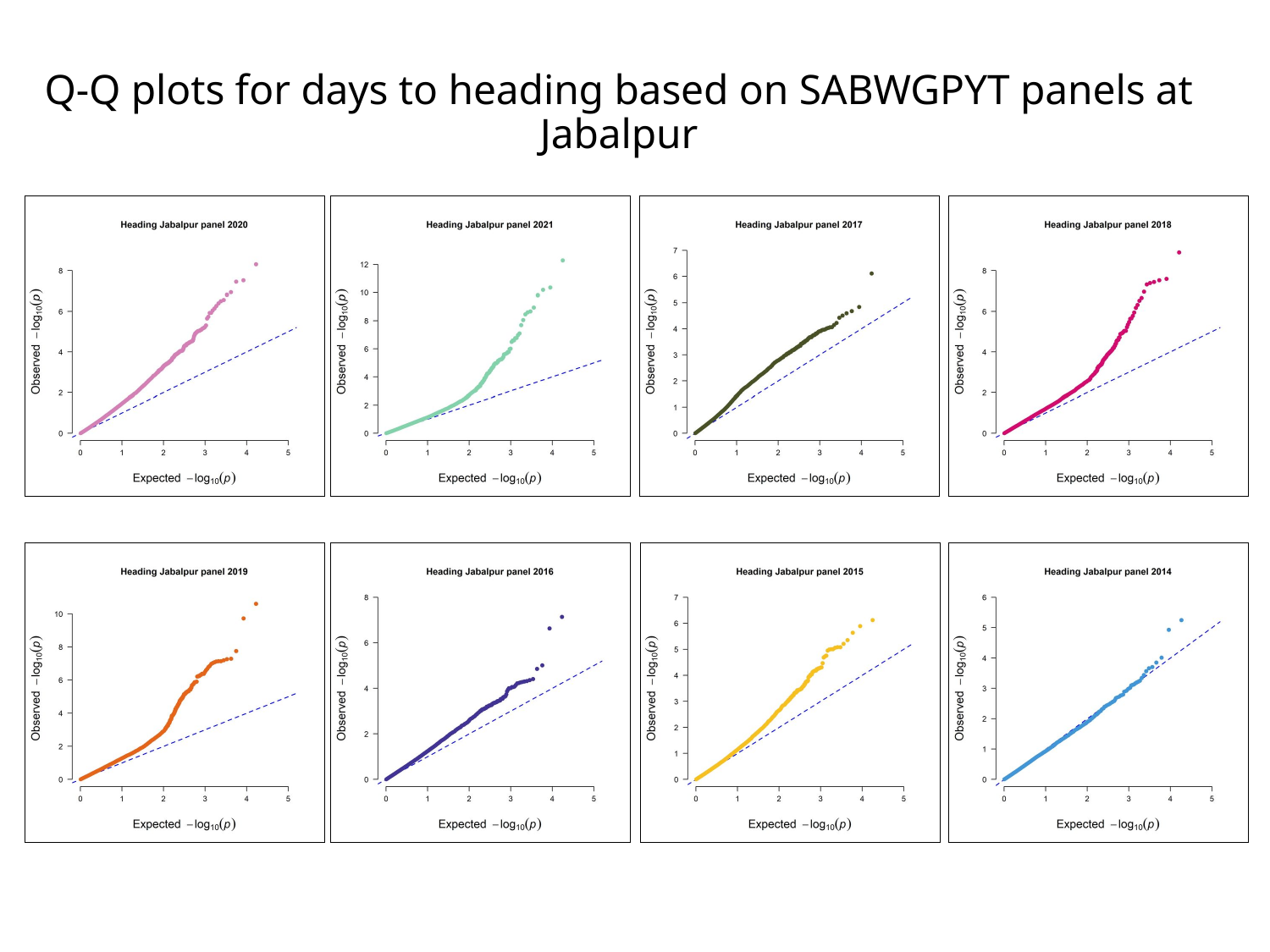

# Q-Q plots for days to heading based on SABWGPYT panels at Jabalpur

## Slide 2
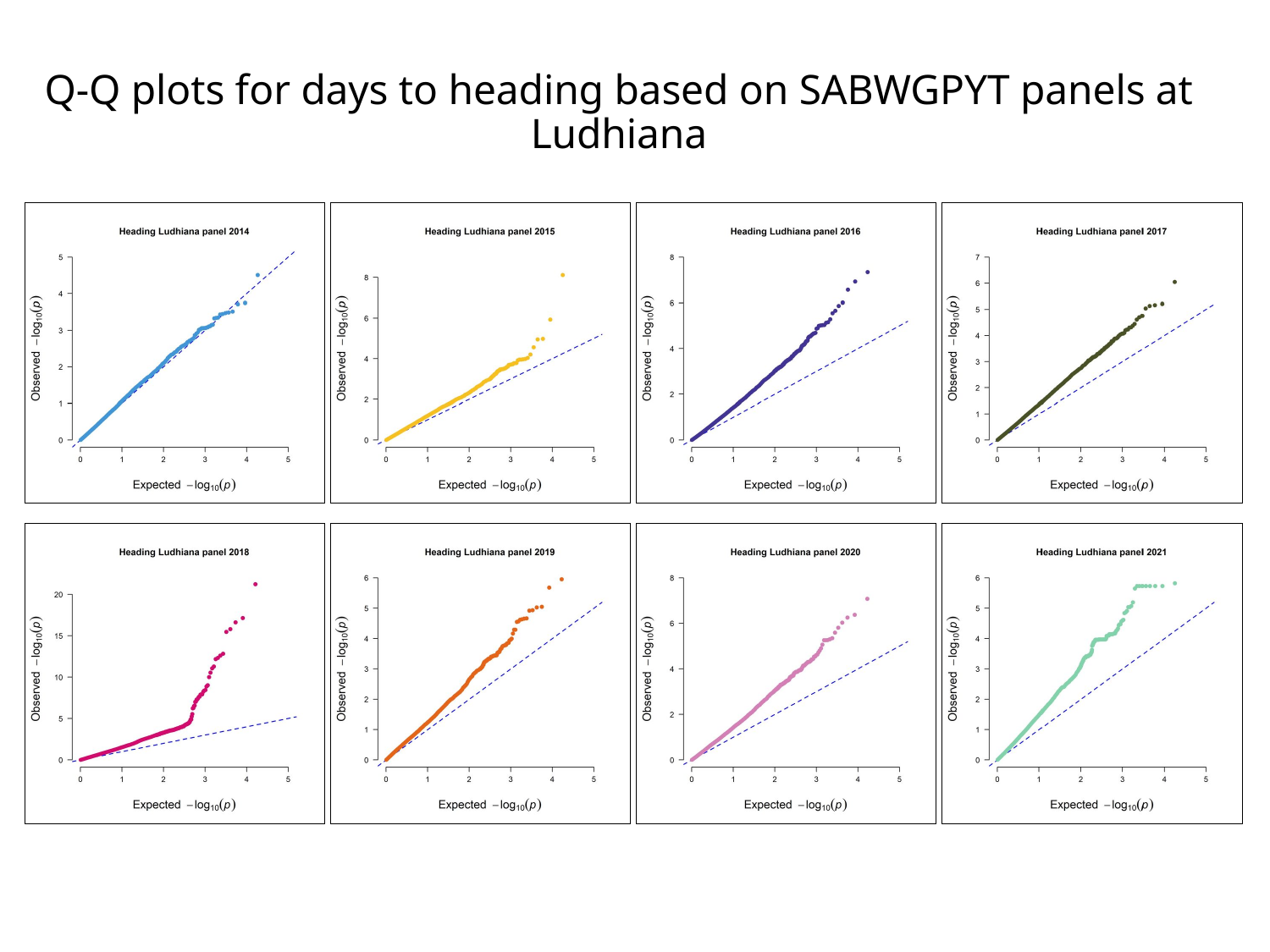

# Q-Q plots for days to heading based on SABWGPYT panels at Ludhiana

## Slide 3
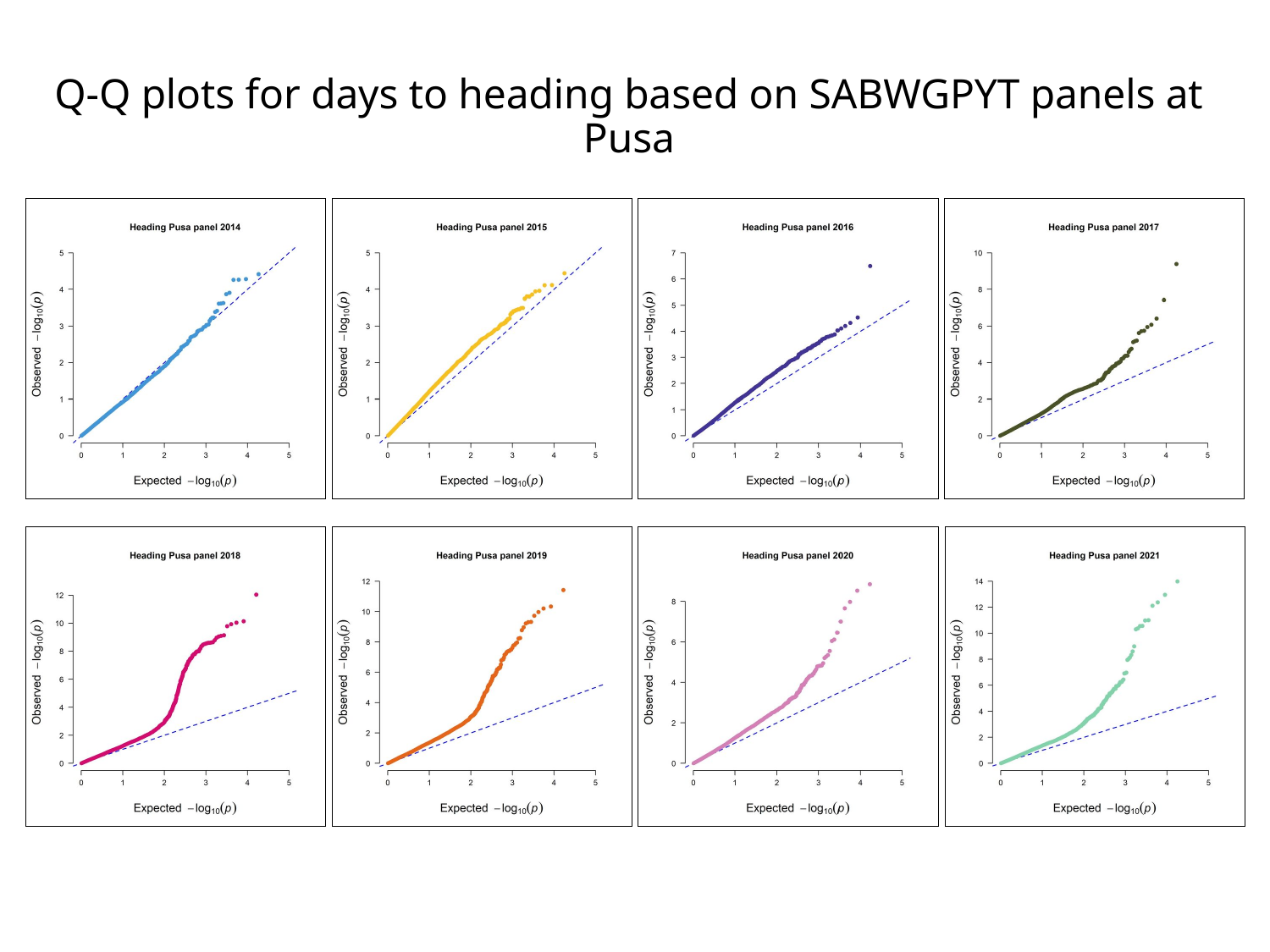

# Q-Q plots for days to heading based on SABWGPYT panels at Pusa

## Slide 4
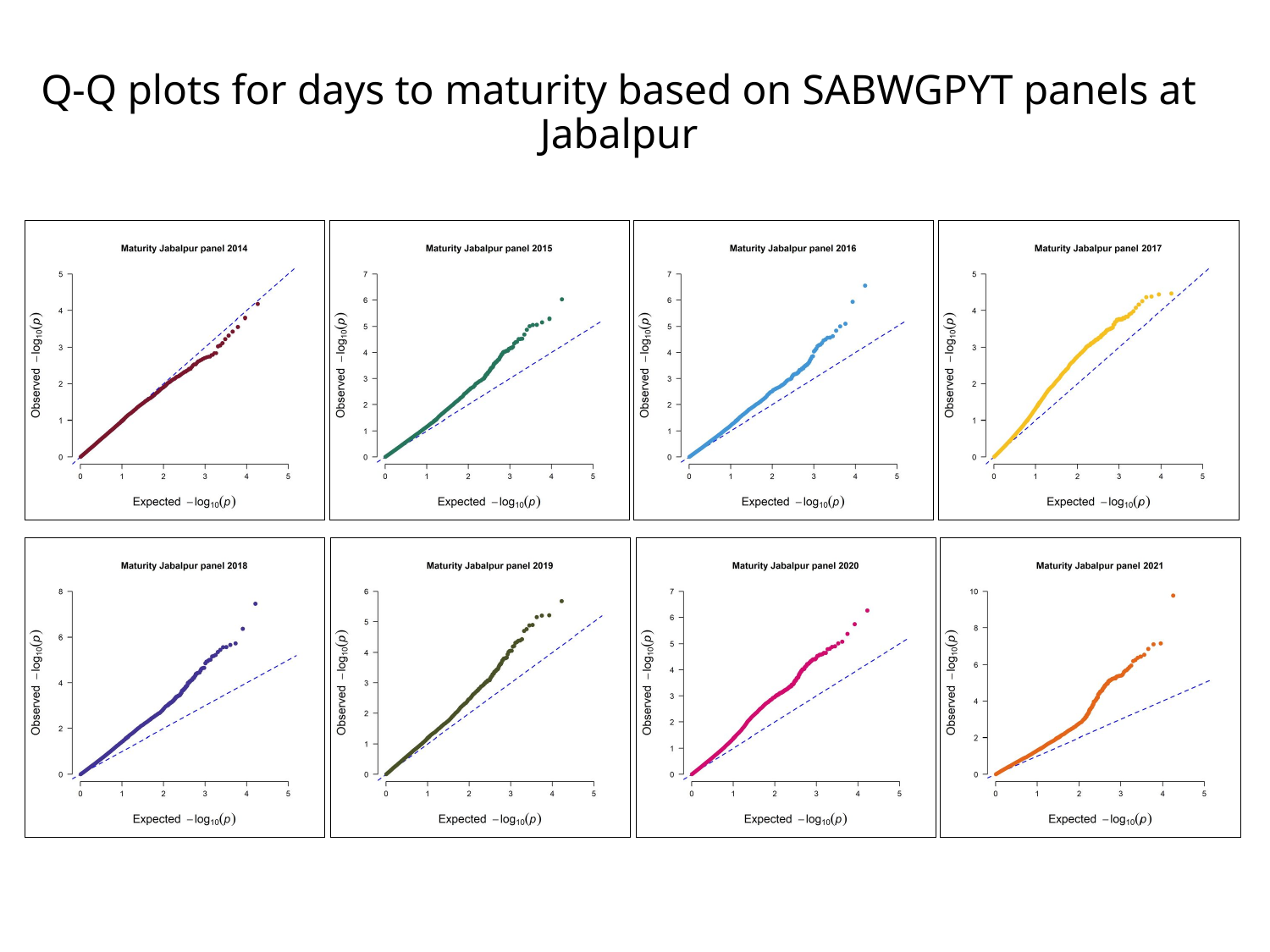

# Q-Q plots for days to maturity based on SABWGPYT panels at Jabalpur

## Slide 5
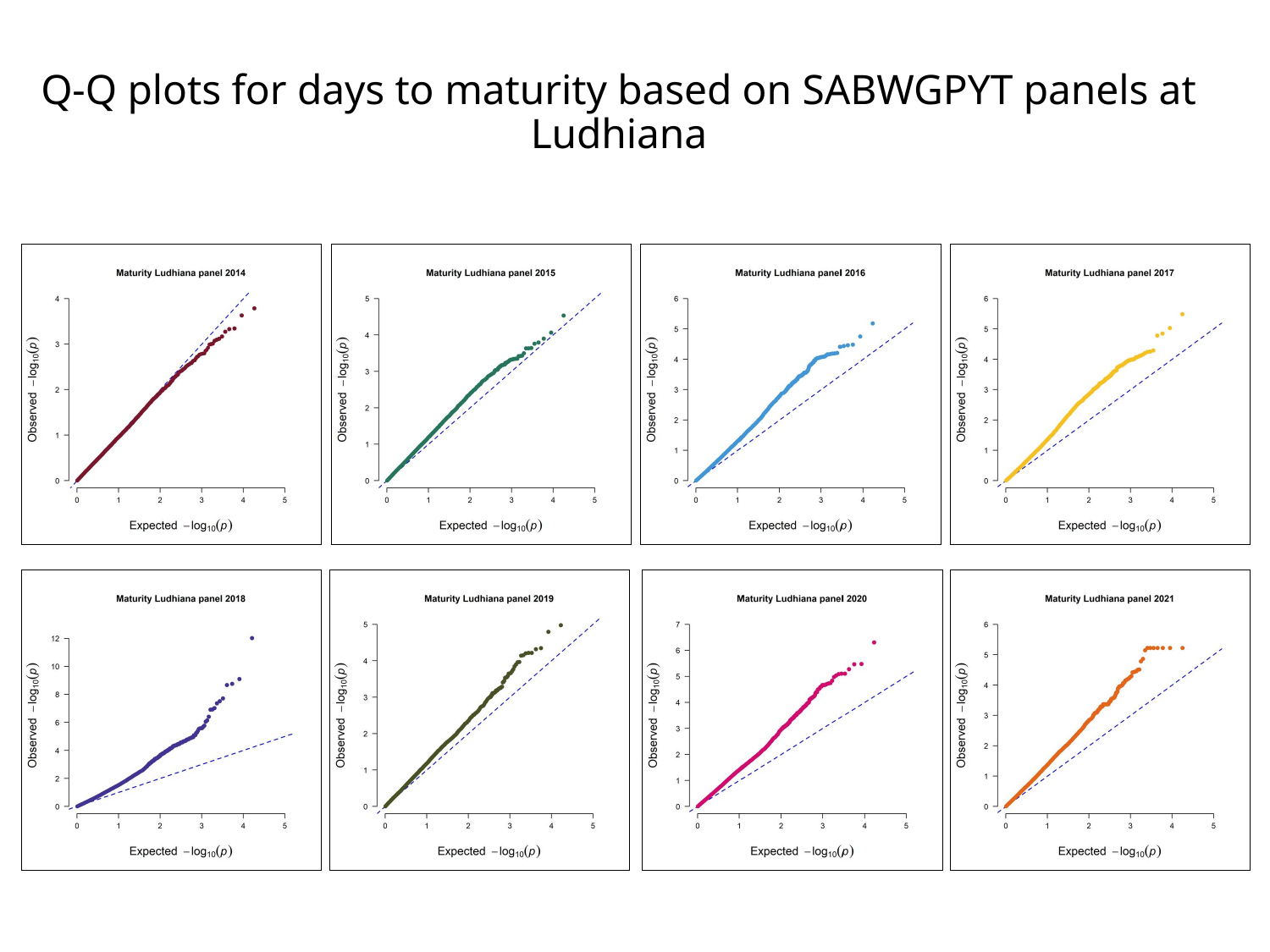

# Q-Q plots for days to maturity based on SABWGPYT panels at Ludhiana

## Slide 6
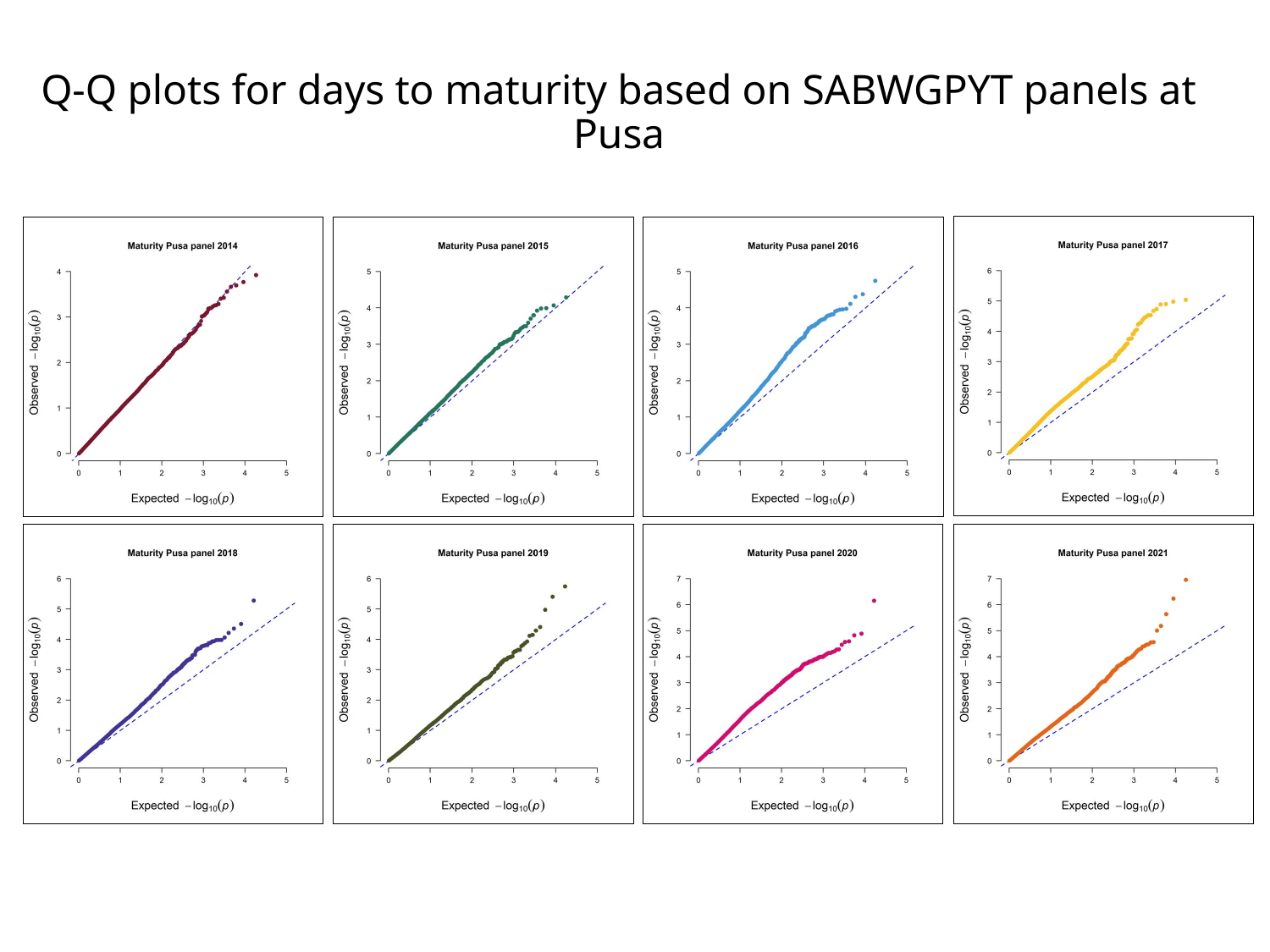

# Q-Q plots for days to maturity based on SABWGPYT panels at Pusa
